# Structural variability and dynamics in the ectodomain of an ancestral-type classical cadherin revealed by AFM imaging

**DOI:** 10.1242/jcs.258388

**Published:** 2021-07-22

**Authors:** Shigetaka Nishiguchi, Hiroki Oda

**Affiliations:** 1 Laboratory of Evolutionary Cell and Developmental Biology, JT Biohistory Research Hall, 1-1 Murasaki-cho, Takatsuki, Osaka 569-1125, Japan; 2 Department of Biological Sciences, Graduate School of Science, Osaka University, 1-1 Machikaneyama-cho, Toyonaka, Osaka 560-0043, Japan; 3 R&D Group, Olympus Corporation, 2-3 Kuboyama-cho, Hachioji-shi, Tokyo 192-8512, Japan

**Keywords:** Cadherin, Cell adhesion, Homophilic interactions, Molecular structure, Atomic force microscopy, Evolution

## Abstract

Type III cadherin represents the ancestral form of classical cadherin in bilaterian metazoans. *Drosophila* possesses type III and type IVa cadherins, known as DN- and DE-cadherins, respectively. Mature DN- and DE-cadherins have 15 and 7 extracellular cadherin domain (EC) repeats, respectively, with DN-cadherin EC6–EC11 homologous to DE-cadherin EC1–EC6. These EC repeats contain predicted complete or partial Ca^2+^-free inter-EC linkers that potentially contribute to adhesion. Comparative structure–function studies of DN- and DE-cadherins may help us understand the ancestral and derived states of classical cadherin-mediated adhesion mechanisms. Here, using bead aggregation assays, we found that DN-cadherin EC1–EC11 and DE-cadherin EC1–EC6 exhibit Ca^2+^-dependent adhesive properties. Using high-speed atomic force microscopy (HS-AFM) imaging in solution, we show that both DN- and DE-cadherin ectodomains share a common morphological framework consisting of a strand-like and a globule-like portion. Furthermore, the DN-cadherin EC repeats are highly variable, flexible in morphology and have at least three bendable sites, one of which is located in EC6–EC11 and can act as a flexible hinge. Our findings provide insights into diversification of classical cadherin-mediated adhesion mechanisms.

This article has an associated First Person interview with the first author of the paper.

## Abstract (precis)

**Summary:** Atomic force microscopy imaging reveals that the ectodomain of an ancestral-type classical cadherin has a flexibly bendable strand-like portion responsible for homophilic adhesion.

## INTRODUCTION

Cadherins are a molecular family that possess two or more tandem repeats of extracellular cadherin domains (ECs) (Hulpiau and van Roy, 2009). Members of this family are found in all metazoans and some non-metazoans (Nichols et al., 2012), and they engage in a range of physiological functions, including cell–cell adhesion, cell sorting, neural wiring, cell polarity regulation and mechanotransduction (Halbleib and Nelson, 2006; Hirano and Takeichi, 2012; Gul et al., 2017). There are more than 10 known cadherin subfamilies, each of which shows specific structural features related to domain composition and organization (Oda and Takeichi, 2011; Nichols et al., 2012; Gul et al., 2017). The structural basis of cadherin functions has been studied using various techniques, including X-ray crystallography, nuclear magnetic resonance spectroscopy (NMR), conventional electron microscopy (EM), cryo-EM and atomic force microscopy (AFM) (Brasch et al., 2019; Boggon et al., 2002; Häussinger et al., 2002; He et al., 2003; Jaiganesh et al., 2018; Nishiguchi et al., 2016). The identified mechanisms, however, highly vary depending on cadherin type.

Classical cadherins, a metazoan-specific subfamily of cadherins, are Ca^2+^-dependent cell–cell adhesion molecules that have an ectodomain responsible for specific trans-homophilic binding, a single-pass transmembrane domain (TM), and a conserved cytoplasmic domain (CP) that interacts with the actin cytoskeleton via catenins (Takeichi, 2014). The cadherin ectodomains bridging the gap between neighboring cells must resist forces that mediate tissue morphogenesis and homeostasis in multicellular systems (Angulo-Urarte et al., 2020; Guillot and Lecuit, 2013; Leckband and de Rooij, 2014; Lecuit and Yap, 2015).

An intriguing feature of the classical cadherins is that their ectodomains exhibit diverse domain composition and organization (Oda and Takeichi, 2011); accordingly, they have been categorized into many different types, including type I, II, III, IVa, and IVb (Hulpiau and van Roy, 2009; Tanabe et al., 2004; Sasaki et al., 2017). Type I and II cadherins, which are specific to the vertebrate/urochordate lineage, are well-studied classical cadherins whose ectodomains have a slightly curved rod-like structure that consists of five consecutive ECs, referred to as EC1 to EC5. Three Ca^2+^ ions are inserted in each inter-EC linker region to rigidify the rod-like structure of the EC repeats. A preferred model for how these 5-EC cadherins mediate homophilic cell-cell adhesion is based on trans-dimerization of the ectodomains via the membrane-distal EC1s of cadherins extended from opposing cells. In contrast, type III cadherins have much larger ectodomains containing more than 14 ECs, two laminin globular domains (LGs) and three cysteine-rich EGF-like domains (CEs). This cadherin type has been suggested to represent the ancestral form of classical cadherin in bilaterians (Oda et al., 2005). Type IVa/IVb cadherins, specific to the insect/crustacean lineage, have seven (for type IVa) or nine (for type IVb) ECs and one LG. Evidence from genome-based comparative studies suggests that type I/II and type IVa/IVb cadherins are likely to have independently evolved from type III cadherin through lineage-specific domain losses (Oda et al., 2005; Sasaki et al., 2017). Type III cadherins, found in extant bilaterians, possibly evolved from a larger cadherin, similar to those found in extant non-bilaterian metazoans (Chapman et al., 2010; Hulpiau and Van Roy, 2010). Elucidating the mechanisms of adhesion mediated by the ancestral-type classical cadherin is important for understanding the diversification of classical cadherin ectodomain structures through various reductive changes.

**Fig. 1.**
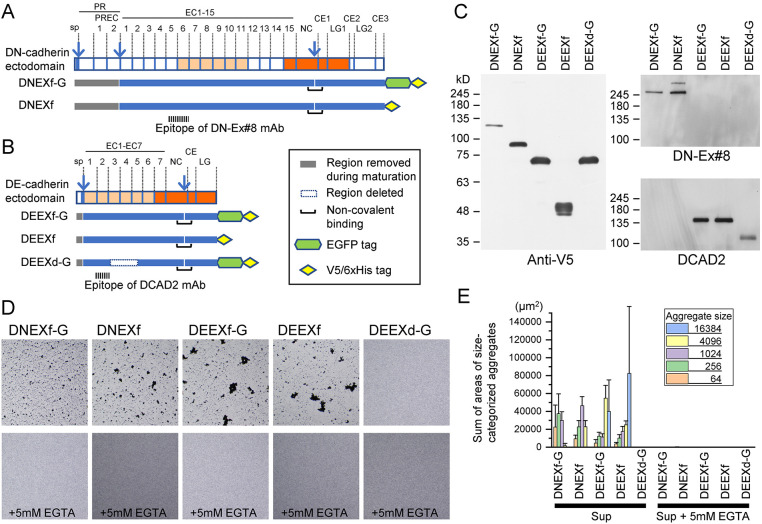
**Bead aggregation assay using full-length ectodomains of DN- and DE-cadherin.** (A,B) Schematic representation of the domain structures of DN-cadherin (A) and DE-cadherin (B) ectodomains, together with their full-length or partially deleted ectodomain constructs. PR, prodomain; EC, extracellular cadherin domain; NC, nonchordate classical cadherin domain; CE, cysteine-rich EGF-like domain; LG, laminin globular domain. Vertical arrows indicate the sites of proteolysis for maturation. The regions of homology between DN- and DE-cadherins are highlighted in light and dark orange (Oda et al., 2005). Feature elements of the constructs are summarized in the inset. The cadherin regions retained and removed during maturation are indicated by blue and gray lines, respectively. The cleaved mature products are non-covalently bound to each other. ‘f’ and ‘d’ in the construct names denote ‘full-length’ and ‘deleted’, respectively. All constructs had a V5/6xHis tag at the C-termini, and three of them had an additional GFP tag between the cadherin region and the V5/6xHis tag, which is denoted by ‘-G’ in their names. DEEXd-G has a deletion in the ECs necessary for homophilic binding (Oda and Tsukita, 1999) and served as control in bead aggregation assays. (C) Western blot analysis of products derived from the cadherin constructs in conditioned medium used for bead aggregation assay. The epitope locations of the monoclonal antibodies (mAb) DN-EX#8 and DCAD2 are indicated in A and B, respectively. An uncleaved precursor product of DNEXf stained with anti-V5 and DN-Ex#8 is visible (see Fig. S1 for details). (D) Images (1886 µm×1886 µm) showing the bead aggregation assay results in the absence (upper) or presence (lower) of 5 mM EGTA. Microbeads conjugated with anti-His-tag antibody were rotated at 150 rpm for 10 min in the conditioned medium. (E) Quantification of the degree of bead aggregation. Individual aggregates were categorized into five classes based on size (inset). The sums of areas of size-categorized aggregates were calculated. Three to six independent transfections were performed for each construct. Graph represents mean±s.d.

*Drosophila* possesses type III and type IVa cadherins, which are known as DN-cadherin (also known as CadN) and DE-cadherin (also known as Shg), respectively. Comparative studies of these cadherins may help us understand the ancestral and derived states of classical cadherin-mediated adhesion mechanisms and the transition from the former to the latter. The mature DN-cadherin ectodomain consists of, in order, 15 ECs (EC1–EC15), a non-chordate classical cadherin domain (NC), a cysteine-rich EGF-like domain 1 (CE1), laminin globular domain 1 (LG1), CE2, LG2 and CE3 (Fig. 1A). The mature DE-cadherin ectodomain consists of, in order, seven ECs (EC1 to EC7), an NC, a CE, and an LG (Fig. 1B). DN-cadherin EC6–EC11 and EC15–LG1 are homologous to DE-cadherin EC1–EC6 and EC7–LG, respectively (Oda et al., 2005; Sasaki et al., 2017). DE-cadherin EC1–EC6 is capable of mediating homophilic cell–cell adhesion in cultured cells and tissues *in vivo* (Haruta et al., 2010). High-speed AFM (HS-AFM) imaging of this DE-cadherin region revealed a tadpole-like morphology, where the head portion exhibits bending at or around the EC2–EC3 linker, which lacks Ca^2+^-binding residues (Jin et al., 2012; Nishiguchi et al., 2016). This Ca^2+^-free state of the EC2–EC3 linker is conserved in type IVa cadherins of multiple insect/hexapod species. Importantly, type IVa cadherin EC2–EC4 can act as determinants for species-specific homophilic binding (Nishiguchi et al., 2016). The DN-cadherin EC7–EC8 linker, which is homologous to the type IVa cadherin EC2–EC3 linker, is predicted to be a partial Ca^2+^-free linker (Jin et al., 2012). The DN-cadherin 3-EC region encompassing this linker is capable of mediating specific binding to DN-cadherin (Nishiguchi et al., 2016).

Bendability and flexibility of EC repeats at complete and partial Ca^2+^-free inter-EC linkers were originally suggested by X-ray crystallography, EM, and molecular dynamics simulation studies of DN-cadherin EC2–EC3 and some EC repeats in several non-classical cadherins (Jin et al., 2012; Tsukasaki et al., 2014; Tariq et al., 2015; Harrison et al., 2016; Powers et al., 2017). However, no studies have provided direct visualization of the structural dynamics and flexibility of EC repeats or evidence for the involvement of bent or folded conformations of EC repeats in adhesion processes. The abovementioned circumstantial evidence on DN- and DE-cadherins suggests that they may be candidates for investigating possible structural and mechanical contributions of EC repeats with complete or partial Ca^2+^-free linkers to cell–cell adhesion.

In the present study, we aimed to provide a foundation for comparative structure–function studies of DN- and DE-cadherin ectodomains in cell-free systems. We used bead aggregation assays to determine the cadherin regions responsible for trans-homophilic binding. We also used HS-AFM to analyze the morphological features of DN- and DE-cadherin ectodomains. Our findings provide new clues for understanding the diversification of classical cadherin-mediated adhesion mechanisms.

## RESULTS

### Soluble DN- and DE-cadherin ectodomains recapitulate Ca^2+^-dependent adhesive properties in cell-free conditions

To investigate the properties and capabilities of the DN- and DE-cadherin ectodomains, we prepared DN- and DE-cadherin cDNA constructs (Fig. 1A,B), with which *Drosophila* S2 cells were transfected to express the DN- and DE-cadherin full-length extracellular regions tagged with a V5/6×His tag (referred to as DNEXf and DEEXf, respectively) or an enhanced GFP/V5/6×His tag (referred to as DNEXf-G and DEEXf-G, respectively) at the C-termini under the control of an actin promoter (Fig. 1C). As a control, we prepared a DE-cadherin construct with a deletion in EC3–EC5, which is essential for cell–cell binding function (Oda and Tsukita, 1999), and a GFP/V5/6×His tag (DEEXd-G; Fig. 1B). Western blot analysis of cell culture supernatants using monoclonal antibodies against the V5 tag, DN-cadherin EC1–EC8 (DN-Ex#8; Iwai et al., 1997) and DE-cadherin EC2 (DCAD2; Oda et al., 1994) showed that mature DN- and DE-cadherin ectodomains were secreted into the medium in soluble form (Fig. 1C). Taking our previous findings into account (Oda and Tsukita, 1999), the expressed DE-cadherin ectodomain was likely to consist of two membrane-distal and -proximal polypeptides that resulted from removal of the signal peptide and proteolytic cleavage in the NC. These polypeptides have been suggested to be bound to each other via the regions flanked by the cleavage site (Oda and Tsukita, 1999). DEEXf-G and DEEXf shared a 145 kDa membrane-distal polypeptide, which was detected with DCAD2. The membrane-proximal polypeptide of DEEXf, but not of DEEXf-G, was detected as multiple signals for unknown reasons. Proteolytic cleavage in the NC was conserved in DN-cadherin, as demonstrated below. DNEXf-G and DNEXf shared a 250 kDa membrane-distal polypeptide, which was detected with DN-Ex#8 (Iwai et al., 1997).

Cell culture supernatants containing DNEXf-G, DNEXf, DEEXf-G, DEEXf and DEEXd-G were examined for their ability to bind microbeads (∼1.6 μm in diameter) conjugated with anti-His-tag monoclonal antibody in the absence or presence of 5 mM EGTA (Fig. 1D). The degree of bead aggregation was numerically evaluated by summing the areas of size-categorized aggregates (Fig. 1E). The relative amount of cadherin molecules in the cell culture supernatants was evaluated by western blotting (Fig. 1C), revealing variations among the constructs. Despite the varying amounts of molecules, all constructs, except for DEEXd-G, induced substantial levels of bead aggregation in a Ca^2+^-dependent manner. These results indicate that soluble DN- and DE-cadherin ectodomains can recapitulate Ca^2+^-dependent adhesive properties under cell-free conditions.

### Multi-step processing of the DN-cadherin ectodomain for maturation

To characterize the multiple polypeptide products of the DN-cadherin ectodomain, we affinity-purified the 250 kDa membrane-distal and 125 kDa membrane-proximal polypeptides of DNEXf-G and then determined their N-terminal 5 amino acid residues, revealing R-V-T-R-A (residues 435–439) and S-P-Y-Y-K (residues 2256–2260), respectively (Fig. S1A,B). The latter sequence indicates conservation of NC cleavage between DE- and DN-cadherin. The former sequence indicates that the DN-cadherin precursor is processed at the junction between the second and third EC repeats, as predicted by computational detection of a furin protease cleavage recognition sequence in a previous study (Jin et al., 2012). The N-terminal region preceding this processing site was designated the prodomain (PR) and the two ECs in the PR were designated PREC1 and PREC2.

To examine the fate of the PR, we prepared two DN-cadherin constructs tagged with a V5/6×His tag, referred to as DNPREC2 and DNEC2 (Fig. S1A). DNPREC2 covered the N-terminal two EC repeats but not the furin-processing site, whereas DNEC2 covered the N-terminal four EC repeats, with the furin-processing site remaining intact. DNPREC2 was used as an antigen to immunize mice and obtain an antiserum (anti-DNPREC2).

To determine whether the N-terminal two EC repeats are retained in the mature DN-cadherin ectodomain, we analyzed the lysates of S2 cells transfected with DNEXf, DNPREC2 and DNEC2, as well as the immunoprecipitates from the cell culture supernatants, with anti-V5-tag antibody (Fig. S1C). The anti-DNPREC2 antiserum recognized specific proteins that corresponded to the longest DNEXf precursor, DNPREC2, and unprocessed DNEC2 in the cell lysates (Fig. S1C, red, brown and light blue asterisks, respectively); however, only the unprocessed DNPREC2 was detected in the immunoprecipitate. Notably, despite the presence of processed derivatives of DNEC4 and DNEXf detected with the anti-V5-tag antibody in both cell lysates and immunoprecipitates (Fig. S1C, light blue triangles), processed derivatives containing the N-terminal two EC repeats were not observed in either the cell lysates or immunoprecipitates. These data suggest that the processed derivative containing the N-terminal two EC repeats is destabilized after the DN-cadherin precursor is processed at the junction between the second and third EC repeats. Therefore, it is most likely that the third EC from the signal peptide is the N-terminal EC of the mature DN-cadherin ectodomain, which was, hence, designated EC1 with the following ECs numbered from 2 to 15.

Furthermore, we performed time-course analysis of the DNEXf-G products in conditioned medium and cell lysates at four different time points after transfection (16 h, 24 h, 48 h and 92 h) by western blotting (Fig. S1D). The primary precursor was the longest product, which was detected with anti-V5, anti-DNPREC2 and DN-EX#8. The second-longest product was a secondary precursor that lacked the PR but possessed an uncleaved NC. The other secondary precursor retained the PR but had a cleaved NC. The former secondary precursor was detected in both cell lysates and conditioned medium, but the latter was detected only in the cell lysates. Moreover, the amounts of fully processed membrane-distal and -proximal polypeptides in the conditioned medium increased with time. These observations indicate that the DN-cadherin ectodomain matures in multiple steps.

### Identification of adhesive units in the DN- and DE-cadherin ectodomains

**Fig. 2.**
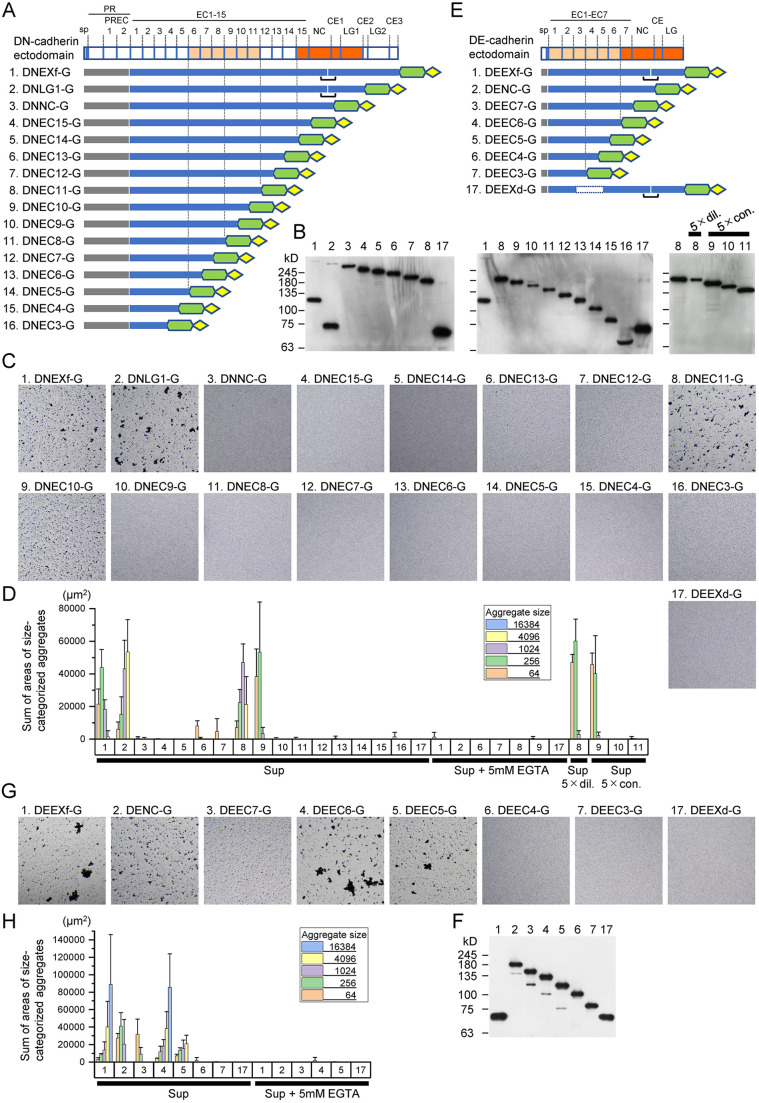
**Bead aggregation assays using DN- and DE-cadherin ectodomain deletion constructs.** (A–H) Datasets from series of DN-cadherin (A–D) and DE-cadherin (E–H) ectodomain deletion constructs are shown separately. (A,E) Schematic representation of series of DN-cadherin (A) and DE-cadherin (E) ectodomain deletion constructs. Feature elements of the constructs are the same as those shown in Fig. 1. The constructs are numbered in each construct series. DEEXd-G served as a negative control, which is numbered 17 in both cases. (B,F) Western blot detection of products from the constructs in conditioned medium using anti-V5-tag antibody. (C,G) Images (1886 µm×1886 µm) showing the bead aggregation assay results using the cell culture supernatants. (D,H) Quantification of the degree of bead aggregation. The sums of areas of size-categorized aggregates were calculated. Bead aggregation assays for selected constructs were performed in the presence of 5 mM EGTA or using cadherin products diluted or concentrated by a factor of 5. Three independent transfections were performed for each construct. Graphs represent mean±s.d.

To narrow down the region responsible for the adhesive property of the DN-cadherin ectodomain, we prepared a series of DN-cadherin ectodomain deletion constructs, which had the same N-termini but different C-termini fused to the GFP/V5/6×His tag (Fig. 2A). These constructs were named DNEC3-G to DNEC15-G, DNNC-G and DNLG1-G after the domain components they covered. The relative amounts of products from the deletion constructs in conditioned medium of transfected S2 cells were examined by western blotting, and were found comparable to each other with the exception of DNEC9-G and DNEC8-G, whose levels were lower than those of the others (Fig. 2B). Bead aggregation assays using supernatants of the conditioned medium revealed that DNEC10-G and DNEC11-G exhibited substantial levels of Ca^2+^-dependent bead–bead binding capabilities, whereas shorter constructs and some of the longer constructs did not (Fig. 2C,D). It is possible, however, that the inability of DNEC9-G and DNEC8-G to induce bead aggregation was due to the lower concentrations of the cadherin products. To examine this possibility, additional bead aggregation assays were performed using DNEC9-G and DNEC8-G products concentrated by a factor of five (Fig. 2B). However, the outcomes were little affected by the concentrations of the cadherin products (Fig. 2D), indicating that the inability of DNEC9-G and DNEC8-G to induce bead aggregation is likely due to their molecular nature. Additional bead aggregation assays using concentrated DNEC10-G and diluted DNEC11-G (Fig. 2D), quantitative comparisons showed that DNEC11-G had a stronger ability to induce bead aggregation than did DNEC10-G. Taken together, these results suggest that DN-cadherin EC1–EC11 constitutes a functional unit that can mediate bead aggregation with substantial strength. In addition, given that some of the constructs longer than DNEC11-G did not induce similarly large aggregates, the function of the EC1–EC11 may be negatively affected by some factors.

The functional unit in the DN-cadherin ectodomain was further evaluated using conventional cell aggregation assays. Various deletions of DN-cadherin ectodomains that corresponded to DNEC8-G, DNEC9-G, DNEC10-G, DNEC11-G, DNEC12-G, DNEC13-G, DNEC14-G and DNEC15-G were fused to the DE-cadherin TM and CP with the addition of a GFP tag (Fig. S2). Cell aggregation assays using S2 cells expressing these chimeric cadherins showed that only the DNEC15-G and DNEC11-G counterparts were capable of inducing cell aggregates. These results suggest that DN-cadherin EC1–EC11, and not a shorter region, constitutes an adhesive unit that can function in both cell-free and cell-based systems.

To facilitate comparative understanding of the domain components responsible for homophilic binding in the DN- and DE-cadherin ectodomains, we also prepared a series of DE-cadherin ectodomain deletion constructs tagged with a GFP/V5/6×His tag (Fig. 2E). These constructs were named DENC-G and DEEC3-G to DEEC7-G after the domain components they covered. The medium supernatants of S2 cells transfected with these constructs contained comparable amounts of the cadherin products (Fig. 2F). In bead aggregation assays using these supernatants, DEEC6-G and DEEC5-G exhibited substantial levels of Ca^2+^-dependent bead–bead binding capabilities, whereas shorter constructs did not (Fig. 2G,H). As was the case with DNEC11-G and DNEC10-G, DNEC6-G induced larger aggregates than did DNEC5-G, and the size distributions of bead aggregates indicated that the adhesion strength of DEEC6-G was comparable to that of DEEXf-G. The longer DEEC7-G showed markedly reduced bead–bead binding capabilities. A similar DE-cadherin ectodomain structure–function relationship was shown in a previous cell-based study (Haruta et al., 2010). Thus, data from the present cell-free bead aggregation assays strengthened the notion that DE-cadherin EC1–EC6 constitutes an adhesive unit.

**Fig. 3.**
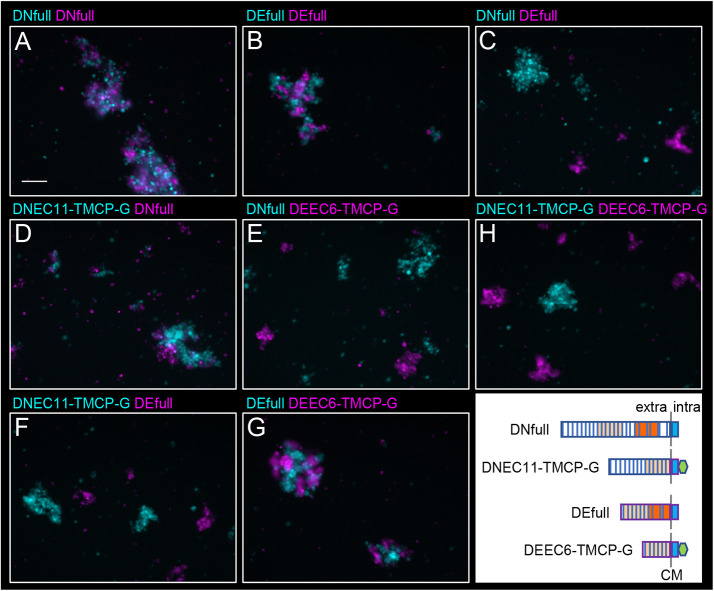
**The identified adhesive units in the DN- and DE-cadherin ectodomains exhibit their original adhesion specificities.** (A–H) Mixed cell aggregation assays using two different cell populations expressing cadherin constructs indicated in cyan and magenta colors. The cells were marked by expression of TagBFP (cyan) or mKate2 (magenta). The inset shows a schematic of the cadherin constructs expressed. The regions derived from DE-cadherin are outlined by purple lines, and those from DN-cadherin by blue lines. The cell membrane (CM) and the extracellular (extra) and intracellular (intra) sides are indicated. The domain components are shown as in Fig. 1. Cells expressing DNfull and DEfull co-aggregated with those expressing the same cadherins (A,B) but separately aggregated from those expressing different cadherins (C). Cells expressing DNEC11-TMCP-G co-aggregated with those expressing DNfull (D) but separately aggregated from those expressing DEfull and DEEC6-TMCP-G (F,H). Cells expressing DEEC6-TMCP-G co-aggregated with those expressing DEfull (G) but separately aggregated from those expressing DNfull and DNEC11-TMCP-G (E,H). Images shown are representative of two experiments. Scale bar: 50 μm.

To examine the binding specificities of the adhesive units identified in the DN- and DE-cadherin ectodomains, we performed mixed cell aggregation assays. Cells expressing the DN-cadherin adhesive unit fused to DE-cadherin TM and CP with a GFP tag (DNEC11-TMCP-G) were co-aggregated with those expressing full-length DN-cadherin (DNfull) but separately aggregated from those expressing full-length DE-cadherin (DEfull) (Fig. 3A–C,D,F). Conversely, cells expressing the DE-cadherin adhesive unit fused to the GFP-tagged DE-cadherin TM/CP (DEEC6-TMCP-G) were co-aggregated with those expressing intact DEfull but separately aggregated from those expressing DNfull (Fig. 3A–C,E,G). Moreover, cells expressing DNEC11-TMCP-G and those expressing DEEC6-TMCP-G formed separate aggregates (Fig. 3H). These results suggest that the identified adhesive units in the DN- and DE-cadherin ectodomains exhibit their original binding specificities.

### AFM imaging of purified DN- and DE-cadherin ectodomains in solution

**Fig. 4.**
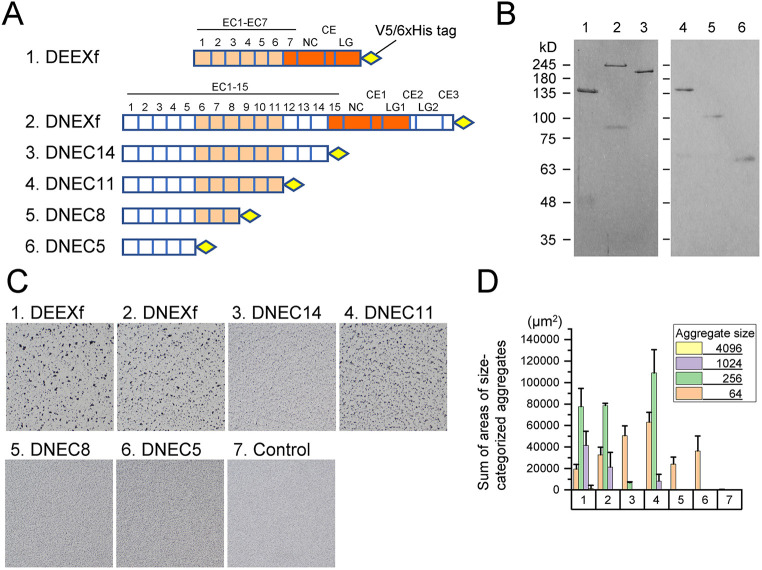
**Bead aggregation assays using purified DE- and DN-cadherin ectodomains.** (A) Schematic representation of the mature DE- and DN-cadherin ectodomain products analyzed (1, DEEXf; 2, DNEXf; 3, DNEC14; 4, DNEC11; 5, DNEC8; 6, DNEC5). The regions of homology between DN- and DE-cadherins are highlighted in light and dark orange. (B) SDS-PAGE separation and Coomassie Brilliant Blue staining of the purified products. (C) Images (1886 μm×1886 μm) showing the bead aggregation assay results using the purified DE- and DN-cadherin ectodomain products at a concentration of 1 nM in HCM buffer containing 1% BSA. Control experiments, numbered 7, were performed similarly but with no cadherin products. (D) Quantification of the degree of bead aggregation. The sums of areas of size-categorized aggregates were calculated. Three independent aggregation assays were performed for each sample type. Graph represents mean±s.d.

To confirm whether purified DN- and DE-cadherin ectodomains exhibit adhesive properties in physiological solution compatible with what was shown in AFM imaging, we performed bead aggregation assays using purified DEEXf and DNEXf at a concentration of 1 nM in HCM buffer (20 mM HEPES pH 7.35, 4 mM CaCl_2_, 10 mM MgCl_2_). As expected, bead aggregates were formed in both cases (Fig. 4A–D). Similarly, four kinds of DN-cadherin ectodomain deletion products, DNEC14, DNEC11, DNEC8, and DNEC5 (non-GFP versions of DNEC14-G, DNEC11-G, DNEC8-G and DNEC5-G, respectively), were purified to test their bead aggregate-inducing abilities (Fig. 4A–D). The assay results were consistent with those obtained with their GFP-tagged counterparts in cell culture supernatants.

**Fig. 5.**
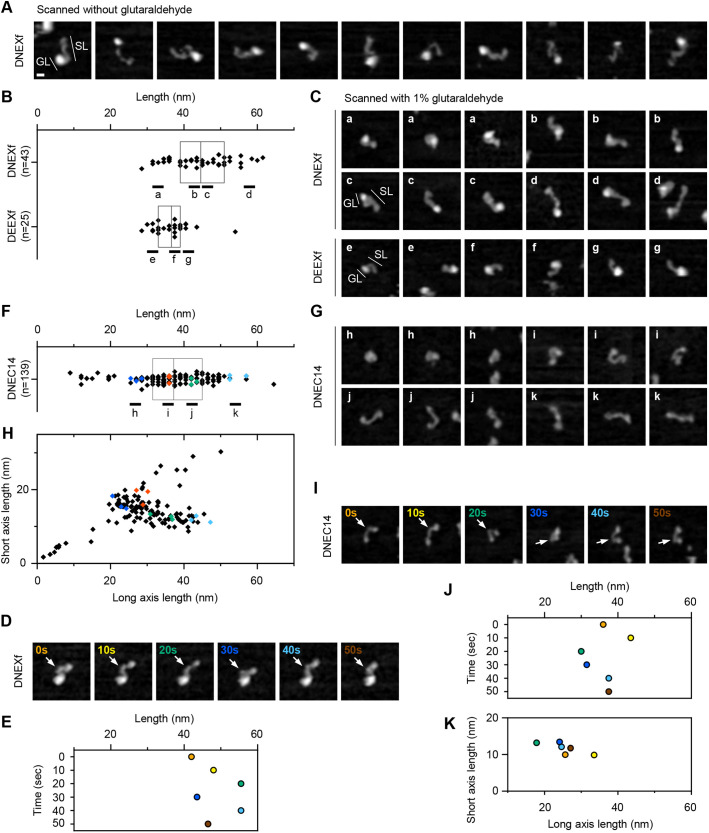
**AFM imaging of purified DN- and DE-cadherin ectodomains.** (A) Image representation of various DNEXf molecules scanned without glutaraldehyde. (B,C) Length measurement and image representation of various DNEXf (*n*=43) and DEEXf (*n*=25) molecules scanned with 1% glutaraldehyde. The length of the imaged object was defined as the maximum distance between two points in the object. The length values are displayed by scattered box plots (B), and the images of molecules from the value ranges indicated in B (a–g) are displayed in C. The boxes represent the middle quartiles with the line between them representing the median. The strand-like (SL) and globule-like (GL) portions are indicated. (D,E) Sequential scanning images showing a single DNEXf molecule (D) and plot showing its length changes (E). (F–H) Dimension measurements and image representation of various DNEC14 molecules. All objects (*n*=139) from 18 selected DNEC14 AFM images were examined. The length values are displayed by scattered box plot (F), and the images of molecules from the value ranges indicated in F (a–g) are displayed in G. Note that no region corresponding to the globule-like portion is observed in DNEC14 molecules. The lateral dimensions of the objects were analyzed by ellipse fitting, with the scattered plot showing the short and long axis lengths (H). (I–K) Sequential scanning images showing a single DNEC14 (H) molecule and plots showing their dimensional changes (J,K). See also Movies 2 and 3. Folding and unfolding, and bending and stretching behaviors of the molecules are captured. The sites marked by white arrows appear to act as a flexible hinge. The dimension values at different time points in D and I are indicated by the colors used in E,J, and K. More examples of DNEC14 molecules are shown in Fig. S3C. Scale bar: 10 nm. All images are displayed at the same scale.

Using HS-AFM in solution, we analyzed the morphological features of the DN- and DE-cadherin ectodomains. Purified DNEXf and DEEXf molecules were adsorbed onto mica substrates in HCM buffer and then imaged by tip scanning. In both cases, most molecules were likely monomers that exhibited asymmetric morphologies, with a distinct globule-like portion on one side of the molecule (Fig. 5A; Fig. S3A), from which a strand-like portion was extended to the other side. However, due to high degrees of fluctuations during tip scanning, the clarity of molecular outlines was limited (Fig. 5A; Fig. S3A,B, Movie 1). The fluctuations were restricted to some extent by replacing the buffer with HCM buffer containing 1% glutaraldehyde prior to tip scanning; this allowed us to acquire reproducible and clearer images of individual DNEXf and DEEXf molecules demonstrating the presence of a strand-like portion linked to a globule-like one in the cadherin ectodomain (Fig. 5B,C; Fig. S3A,B). Hereafter, tip scanning for acquiring AFM images was performed in the presence of 1% glutaraldehyde unless otherwise indicated.

Although the majority of DNEXf and DEEXf molecules appeared to be monomers, there were a few cases (<5%; Fig. S4A,B) where two molecules contacted or overlapped with each other. However, due to technical limitations, it was difficult to prove the specificity and reproducibility of interactions between such molecules.

### Structural variability and dynamics in the DN-cadherin ectodomain

To examine the structural variability of the DN- and DE-cadherin ectodomains, the length of manually selected DNEXf (*n*=43) and DEEXf (*n*=25) molecules was measured, which was defined as the maximum distance between two points in the individual objects (Fig. 5B). Plotting showed that the length varied in each molecule type. Among the DNEXf molecules, folded or extended morphologies were observed depending on the length (Fig. 5B,C). Tracking of the dimension and shape of a single DNEXf molecule revealed length fluctuations that were correlated with bending or folding and stretching or unfolding behaviors of the strand-like portion in the DN-cadherin ectodomain (Fig. 5D,E; Movie 2). Among the DEEXf molecules, the variation in the dimension and shape was less prominent than among DNEXf molecules (Fig. 5B,C).

To investigate the polarity of DNEXf morphology, we imaged DNEC14, which lacks the membrane-proximal region containing two LGs. DNEC14 molecules, in many cases, exhibited rather symmetric morphologies, in which the globule-like portion appeared to be missing or largely reduced (Fig. 5G). This observation indicates that the globule-like portion is located on the membrane-proximal side while the strand-like portion is on the membrane-distal side in the DN-cadherin ectodomain. Our previous work using the same AFM technique showed that DE-cadherin EC1–EC6 is folded to form a tadpole-like structure, which was observed in the strand-like portion of DEEXf in the present work. This further indicates that the globule-like portion is located on the membrane-proximal side in the DE-cadherin ectodomain. Taken together, the results demonstrate that the DN- and DE-cadherin ectodomains share a common morphological framework consisting of the strand-like and globule-like portions, corresponding to the membrane-distal and -proximal regions containing the EC repeats and LG(s), respectively.

To quantitatively describe the structural variation of DNEC14 molecules, we measured the lateral dimensions of all individual objects (*n*=139) collected from 18 selected AFM images for DNEC14 by ellipse fitting, in addition to simply measuring the length (Fig. 5F,H). Plotting the long and short axis lengths revealed large variations indicating that molecules in the middle range showed a tendency for those with large long axis lengths to have small short axis lengths (Fig. 5H). These dimensional variations were correlated with the shape variations (Fig. 5F–H). Consistently, tracking the dimensions of single DNEC14 molecules showed that folding and unfolding behaviors of the molecule using a flexible hinge in the middle fluctuated the long and short axis lengths (Fig. 5I–K; Fig. S3C,D, Movie 3). Taken together, these data demonstrate the structural variability and dynamics of the strand-like portion in the DN-cadherin ectodomain.

### Knot-like and bent or kinked structures in the strand-like portion of the DN-cadherin ectodomain

**Fig. 6.**
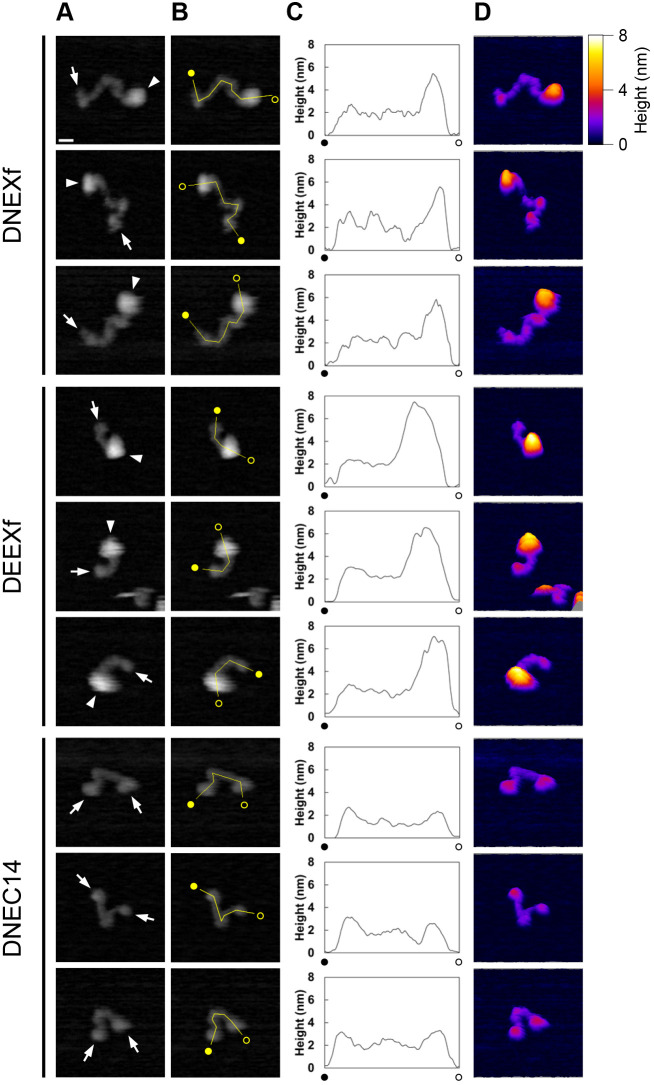
**Comparative morphological characterization of DN- and DE-cadherin ectodomains by HS-AFM at a high resolution.** (A) AFM images showing three different molecules for each of DNEXf, DEEXf and DNEC14. White arrows in the DNEXf and DEEXf molecules indicate a knot-like structure located at or near the end of the strand-like portion opposite to the globule-like portion (white arrowheads). Knot-like structures are present at both ends of the DNEC14 molecules, with a bent structure in the middle. (B,C) Height profiles along the length of the molecules. Filled and open circles indicate the starting and ending points of the lines along which the height values were obtained. (D) Three-dimensional representation of the molecules. The tilt angle is 10°. Pseudo color indicates the height relative to the surface of the mica. Scale bar: 10 nm. All images are displayed at the same scale. Images shown are representative of two or more experiments.

To further characterize the strand-like and globule-like portions of the DN- and DE-cadherin ectodomains, we examined DNEXf, DEEXf and DNEC14 molecules at a high resolution by AFM. Still images showing molecules in which the strand-like portion was loosely folded and the outline was clearly represented were selected (Fig. 6A) and analyzed (Fig. 6B–D). Height mapping and profiling revealed that the maximum height in the globule-like portions of DNEXf and DEEXf was more than ∼5 nm (DNEXf, 5.9 nm, 5.5 nm, 5.8 nm, *n*=3; DEEXf, 6.7 nm, 7.5 nm, 7.3 nm, *n*=3), whereas that in DNEC14 and the strand-like portions of DNEXf and DEEXf was up to ∼3 nm (DNEXf, 3.1 nm, 3.0 nm, 3.5 nm, *n*=3; DEEXf, 3.5 nm, 2.5 nm, 2.9 nm, *n*=3; DNEC14, 2.7 nm, 3.3 nm, 3.2 nm, *n*=3). The globule-like portions were measured to occupy ∼50% of the total volume in DNEXf and ∼70% in DEEXf, respectively. It was previously shown that DE-cadherin EC1–EC4 has a bendable site that contributes to the formation of a small globule-like structure (Nishiguchi et al., 2016), which was distinguished from the LG-containing globule-like portion by size. To avoid confusion, such small-sized globule-like structures in the strand-like portions of cadherin ectodomains were described as knot-like structures. In the height profile, knot-like, bent or kinked structures were recognizable as local maxima. Near the membrane-distal end of the DNEXf strand-like portion, a distinct knot-like structure with a faint tail was observed (Fig. 6A). At more membrane-proximal regions in the DNEXf strand-like portion, additional knot-like, bent or kinked structures were recognized. DNEC14 molecules showed similar-sized knot-like structures at both ends and a bent or kinked structure in the middle (Fig. 5G,I). Together, these observations suggest that there are at least three bendable sites in DN-cadherin EC1–EC14.

### The location of multiple bending sites in DN-cadherin EC1–EC14

To identify the bending site locations in DN-cadherin EC1–EC14, we comparatively analyzed the AFM-determined morphology of DNEC14, DNEC11, DNEC8 and DNEC5. In addition to the abovementioned 149 objects for DNEC14, 180 independent objects for DNEC11, 144 for DNEC8 and 82 for DNEC5 were collected from still AFM images, and their areas were individually measured (Fig. S5). To simplify the comparative analyses in subsequent steps, objects that had an area ranging between mean±1 s.d. were systematically selected for each molecule type (Fig. S5A). The lateral dimensions of all the selected objects were measured using ellipse fitting (Fig. S5B). Plots of the long and short axis lengths showed distributions reflecting the amino acid sequence length but with substantial variations.

**Fig. 7.**
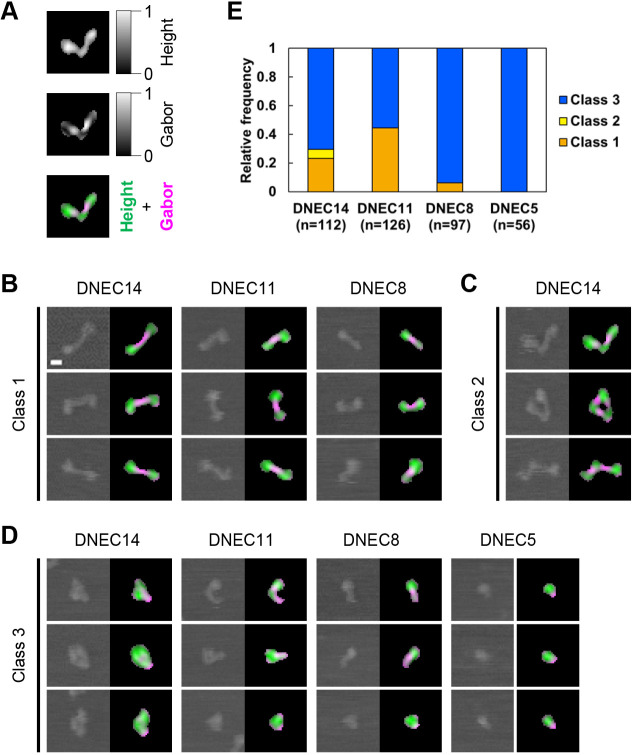
**Comparative morphological characterization of DN-cadherin ectodomain deletion products.** (A) Representative example of AFM image segmentation for DNEC14. The top panel is an image processed by normalization of the height values; the middle panel is an image processed by a rotational series of Gabor filters (Fig. S6; see Materials and Methods); the bottom panel is a merge of the two images using pseudo colors. (B–D) Categorization of objects from AFM images for DNEC14 (*n*=112), DNEC11 (*n*=126), DNEC8 (*n*=97), and DNEC5 (*n*=56) molecules. Representative cases with class 1 (B), class 2 (C), and class 3 (D) morphologies are presented (left, raw images; right, segmented images). All cases are listed in the online data (Nishiguchi and Oda, 2021). (E) Cumulative bar chart showing the relative frequency of cases with class 1, 2, and 3 morphologies for DNEC14, DNEC11, DNEC8, and DNEC5 molecules. Scale bar: 10 nm. All images in A–D are displayed at the same scale.

For objective morphological categorization of AFM-imaged molecular objects, we developed an image segmentation method using a rotational series of two-dimensional Gabor filters. The Gabor filters, which were applied to binarized AFM images, were optimized for positively detecting straight stripes of 6–9 nm in width, corresponding to linear EC repeats, and negatively detecting knotted and bent or kinked regions (see Materials and Methods; Fig. S6). The output of Gabor filter processing was combined with the height image, which highlighted the knotted and bent or kinked regions in individual objects (Fig. 7A). All systematically selected objects for DNEC14, DNEC11, DNEC8, and DNEC5 were processed using this segmentation method.

When investigating the sets of raw and segmented AFM images, we defined three molecular morphology classes 1–3 (Fig. 7B–D) and categorized all the objects into the three classes (Fig. 7B–E; Nishiguchi and Oda, 2021). Class 1 morphology had recognizable knot-like structures at both ends that were linked by a linear rod-like element (Fig. 7B). Similarly, class 2 morphology had knot-like structures at both ends but that were linked by a bent or kinked element (Fig. 7C). Morphologies other than class 1 and 2 were categorized as class 3 morphology (Fig. 7D). In total, 23% and 6% of DNEC14 objects exhibited class 1 and class 2 morphology, respectively (Fig. 7E). However, no class 2 morphology was found in any other molecular type. Moreover, 44% of DNEC11 objects exhibited class 1 morphology, which also occurred in DNEC8 objects, although much less frequently (6%). Neither class 1 nor class 2 morphology was found in DNEC5 objects.

**Fig. 8.**
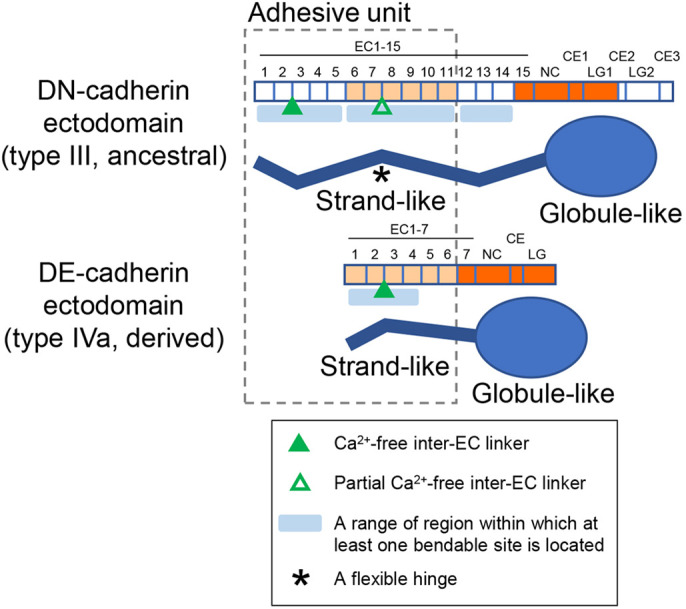
**Comparison of the structural and functional features between the DN- and DE-cadherin ectodomains.** Schematics show the domain structures (upper) and AFM-imaged morphological features (lower) of the DN- and DE-cadherin ectodomains. They share a common morphological framework that consists of the strand-like (zigzag lines) and globule-like (ellipses) portions. DN-cadherin EC1–EC11 and DE-cadherin EC1–EC6, which contain six EC repeats homologous to each other (light orange), constitute adhesive units capable of mediating homophilic adhesion in cell-based and cell-free systems (dashed line box). Each corner of the zigzag lines indicates possible presence of at least one bendable site within each region indicated by light blue bars. Note that the angle of the corners in the zigzag lines has no specific information except for indicating the presence of bendable sites. The bendable site marked by asterisk in the DN-cadherin ectodomain can act as a flexible hinge and is possibly located in the differential region of DNEC5 and DNEC8 (EC6–EC8). The presence of one bendable site in DE-cadherin EC1–EC4 has been demonstrated in previous work (Nishiguchi et al., 2016). Complete (filled triangles) and partial (open triangle) Ca^2+^-free inter-EC linkers have been demonstrated or predicted as shown (Jin et al., 2012).

Morphological categorization of the DN-cadherin ectodomain deletion products provided information on the possible locations of bendable sites in the EC repeats (Fig. 8). The occurrence of class 2 morphology was unique to DNEC14, indicating that DN-cadherin EC1–EC14 has at least three bendable sites. The absence of class 2 morphology in DNEC11 suggests that at least one bendable site is located in the differential region of DNEC14 and DNEC11 (EC12–EC14). The absence of class 1 morphology in DNEC5 suggests that at least one bendable site is located in the differential region of DNEC11 and DNEC5 (EC6–EC11). Most DNEC5 objects were observed in a ball-like shape, indicating that EC1–EC5 contains a knot-like structure observed at or near the membrane-distal end of DNEXf. The observation that some DNEC10 objects exhibited class 1 morphology indicates that DN-cadherin EC1–EC8 has at least two bendable sites mutually separated, implying that one of them is likely to be located within the EC6–EC8.

## DISCUSSION

The present study explored the structure–function relationships of the DN- and DE-cadherin ectodomains, which represent the ancestral (type III) and derived (type IVa) states, respectively, of classical cadherin evolution in arthropods (Oda et al., 2005; Sasaki et al., 2017). Through HS-AFM, we showed that the DN- and DE-cadherin ectodomains share a common morphological framework that consists of a membrane-distal strand-like portion and a membrane-proximal globule-like one, which likely correspond to the EC repeats and non-EC domains, respectively, although the exact morphological boundary between the strand-like and globule-like portions has not been determined. We provided biochemical evidence to suggest that the N-terminal region containing the two EC repeats is removed from the DN-cadherin mature product.

Bead- and cell-based aggregation assays also identified the DN- and DE-cadherin adhesive units, which consist of 11 (EC1–EC11) and six (EC1–EC6) EC repeats, respectively. The latter six EC repeats are homologous to the C-terminal six EC repeats of the former 11 EC repeats (EC6–EC11). These units are separable from the remaining cadherin parts, and mediate the substantial strength and specificity of trans-homophilic binding. An important aspect of our findings was that, in contrast to the rod-like conformation of the 5-EC ectodomain of typical vertebrate classical cadherins, the EC repeats in the DN- and DE-cadherin ectodomains exhibited folded conformations. Notably, we found that morphology of the DN-cadherin EC repeats was highly varied and dynamically changeable, as evidenced by AFM. Comparative analyses of deletion constructs showed that the DN-cadherin EC repeats had at least three bendable sites, with at least two of them located in the adhesive unit and one of the two being flexible and located in EC6–EC11. Folding and unfolding, and flexible bending of the EC repeats, at least in part, account for the structural variability in the DN-cadherin ectodomain; however, the functional relevance of such structural variability and dynamics could not be explored in the present work.

### Precursor processing for classical cadherin maturation

Both DN- and DE-cadherin ectodomains undergo proteolytic cleavage at a conserved site in the NC. Sequence conservation at the corresponding site is found in most known non-chordate classical cadherins, suggesting an ancient origin of this proteolytic cleavage in classical cadherin evolution. However, no functional significance of NC cleavage has been revealed, as animals in which endogenous DE-cadherin is replaced with a mutated DE-cadherin devoid of NC cleavage can survive with no detectable defects (Haruta et al., 2010).

Removal of the N-terminal prodomain for DN-cadherin maturation resembles the precursor processing for type I/II cadherins in vertebrates, which involves furin or other proprotein convertase (Ozawa and Kemler, 1990; Posthaus et al., 1998). The precursor regions of type I/II cadherins have structural similarities to ECs, although these similarities are low at the sequence level (Koch et al., 2004). It is possible that this processing mode of type I/II cadherins was inherited from an ancestral cadherin, such as type III cadherin. In contrast to the cases of type I/II cadherins, however, one of the ECs in the DN-cadherin prodomain retains conserved Ca^2+^-binding residues, raising questions about its potential function. Time-course analysis of products from DNEXf-G showed that removal of the prodomain and NC cleavage appeared to occur in parallel rather than in a specific order. However, precursor products containing the prodomain did not appear in the culture medium of S2 cells transfected with DN-cadherin ectodomain constructs, whereas precursor products without NC cleavage did appear. These observations led us to speculate that removal of the prodomain is a prerequisite for DN-cadherin to expose a functional ectodomain to the outside of the cell. This is not the case for NC cleavage. The observation that DE-cadherin as well as other type IV cadherins has no region corresponding to the DN-cadherin prodomain (Nishiguchi et al., 2016; Sasaki et al., 2017) indicates evolutionary modifications in the processing steps for classical cadherin maturation.

### Possible mechanisms of trans-homophilic binding by ancestral-type classical cadherin

We conducted bead-based aggregation assays to quantify the adhesive properties of various DN- and DE-cadherin ectodomain fragments, most of which were tagged with GFP. Substantially different sizes of bead aggregates formed by DNEXf-G and DNEXf (Fig. 1D,E) might indicate that the addition of a GFP tag has a negative effect on adhesion capability. However, since our western blot data (Fig. 1C) showed that the amount of DNEXf-G was less than that of DNEXf, the addition of a GFP tag might have affected the efficiency of protein synthesis or the stability of the product. Therefore, we also considered the possibility that the smaller aggregates formed by DNEXf-G could be due to a lower concentration of cadherin molecules. In contrast, similar sizes of aggregates were formed by similar amounts of DEEXf-G and DEEXf (Fig. 1C–E). To enable a comparison of the adhesion properties of different constructs in bead aggregation assays, we checked and presented the amount of cadherin molecules by western blotting, in parallel.

Our cell-free bead aggregation assays showed that the N-terminal 10 EC repeats (EC1–EC10) and five EC repeats (EC1–EC5) of mature DN- and DE-cadherins, respectively, are the minimum requirements for mediating Ca^2+^-dependent trans-homophilic binding. Addition of EC11 (for DN-cadherin) and EC6 (for DE-cadherin) enhanced the binding capacity to levels comparable to those of the full-length ectodomain constructs. Accordingly, based on our results, we defined EC1–EC11 and EC1–EC6 as the adhesive units in the DN- and DE-cadherin ectodomains, respectively. The addition of more ECs to the minimal functional construct, however, led to negative effects on binding ability, as observed in the bead aggregation assays with DNEC12-G, DNEC13-G, DNEC14-G, DNEC15-G and DEEC7-G (Fig. 2). These negative effects were neutralized by further addition of the membrane-proximal domains. There are two mutually nonexclusive possibilities to explain the binding ability variations among the constructs despite all having the adhesive unit. First, the disabled constructs could have an improper orientation in representing the adhesive unit on the surface of the bead or cell. This interpretation is based on our AFM data, which suggest that one of the bending sites in the EC repeats is localized in the differential region of DNEC11 and DNEC14. Second, the ECs that are more membrane-proximal than the adhesive unit might have interactions with the following non-EC domains to constitute another functional and/or structural unit. The lack of a part, not the whole, of this unit might produce a negative effect on the performance of the adhesive unit. These possibilities should be investigated in future studies.

In a previous study by Jin et al. (2012), sedimentation equilibrium analytical ultracentrifugation (SE-AUC) revealed the dimer formation of DN-cadherin EC1–EC9 and EC1–EC10 fragments expressed in human cell lines, while EC1–EC8 fragments were monomers. However, the authors did not provide any evidence for the involvement of the dimers in cadherin trans interactions. Our efforts to directly visualize DN- and DE-cadherin ectodomain dimers or oligomers were fruitless, although we noticed very few potential dimers (Fig. S4). One possible reason for this failure is that the homophilic binding affinity of the molecules is too weak. Another possibility is that the AFM cantilever prevents dimer formation during scanning. In our bead aggregation assays, the surface of the anti-tag antibody-conjugated beads served as an immobilizing carrier, where the tagged ectodomain fragments were condensed and oriented. In contrast, during our sample preparation for AFM, a dilution step was required prior to adsorption onto mica and no specific strategy was adopted to control the density and orientation of the molecules. As bead-based experiments are incompatible with HS-AFM, technical difficulties in direct visualization of low-affinity trans interactions between cadherins should be overcome in future studies.

The presence of at least three bendable sites in DN-cadherin EC1–EC14 explains why the large ectodomain can be accommodated in the limited extracellular space of cell–cell adherens junctions. Bending and kinking of EC repeats have been documented in the Ca^2+^-free DN-cadherin EC2–EC3 linker (Jin et al., 2012) as well as in complete or partial Ca^2+^-free inter-EC linkers in non-classical cadherins (Tsukasaki et al., 2014; Tariq et al., 2015; Powers et al., 2017). However, no such structural features have been linked to trans interactions between opposing cadherins. Our investigations focused on the EC repeats homologous between DN- and DE-cadherins (Nishiguchi et al., 2016) suggest potential cases where bending and kinking of EC repeats may be involved in homophilic cadherin interactions. DN-cadherin EC7–EC8 and DE-cadherin EC2–EC3 linkers, partially and completely lacking Ca^2+^-binding residues, respectively, were predicted to be evolutionarily conserved bendable sites (Fig. 8; Jin et al., 2012). DE-cadherin EC2–EC4 and DN-cadherin EC7–EC9 were shown to contain major determinants of trans-homophilic binding specificities (Nishiguchi et al., 2016). Moreover, HS-AFM revealed bending of EC repeats at or near the predicted sites in both cadherins. The bending of EC repeats in the middle of DN-cadherin EC1–EC14, which was possibly located in the differential region of DNEC5 and DNEC8 (EC6–EC8), was highly flexible (Fig. 8), providing the first example of flexibly bent cadherin ectodomain conformations directly visualized in solution. This visualization was achieved by HS-AFM, highlighting the potential of this technology for studying the dynamics of cadherin structures. The bending of the corresponding EC repeats in DE-cadherin appeared to be more stable, adopting a knot-like morphology at the membrane-distal region (Nishiguchi et al., 2016). The bent conformations of EC repeats observed in the DN- and DE-cadherin adhesive units might be part of the adhesion mechanisms. The flexible nature of the EC repeats might be associated with the possible creation of ‘open’ and ‘closed’ states of molecular surfaces available for protein–protein interactions under tension (Pruitt et al., 2014) as well as conferring plasticity to adhesion assembly (Tariq et al., 2015; Harrison et al., 2016; Powers et al., 2017). The HS-AFM technique can also potentially be used to analyze structural dynamics of cadherin ectodomains associated with their adhesive functions, as applied in studies on force generation and dynamics of cytoskeletal components in reconstituted systems (Kodera et al., 2010; Fujita et al., 2019).

The presence of membrane-proximal extracellular regions containing CEs and LGs is a structural feature shared by classical cadherins and two other cadherin subfamilies, Fat and Celsr (also known as Flamingo), although vertebrate/urochordate type I/II cadherins are the exception (Oda and Takeichi, 2011). The AFM data on DN- and DE-cadherin ectodomains provide morphological information on such non-EC regions of cadherins, revealing a distinct globule-like morphology. Despite the differences in the number of the constituent domains, however, we were not able to recognize differences in size and shape between the DN- and DE-cadherin globule-like portions. This may be due to insufficient resolution of AFM or other unknown reasons. There were multiple cases in which two DE-cadherin ectodomains were closely tied with each other via the globule-like portions; thus the possibility of cis interactions between the globule-like portions should be investigated. Moreover, genetic evidence suggests that the DE-cadherin membrane-proximal extracellular region is required for apical constriction driven by actomyosin contraction during epithelial bending (Martin et al., 2009; Haruta et al., 2010). It is thus reasonable to consider that the cadherin globule-like portions play supportive roles in force-resisting homophilic adhesion.

Finally, why the ancestral-type classical cadherin is extraordinarily large; why various reductive changes in classical cadherin domain organization were permissive during animal evolution, despite the structural conservation within some other cadherin subfamilies (Oda and Takeichi, 2011); and what kind of impact such changes had on the mechanical and dynamical properties of the cell-cell adhesion interfaces are intriguing questions. Our technical applications and findings provide new avenues for tackling these questions.

## MATERIALS AND METHODS

### DNA construction

To express DE- and DN-cadherin ectodomains in S2 cells, the expression plasmid pAc5.1/V5-His A (Thermo Fisher Scientific, Waltham, MA, USA) was used. To prepare DNA constructs for expression of EGFP-tagged ectodomains, an EGFP-coding fragment amplified by PCR was transferred to pAc5.1/V5-His A from pAcHis-DEEC6-EGFP-His (Nishiguchi et al., 2016) using *Xho*I and *Pme*I restriction sites; this plasmid was designated as pAcHis-EGFP. DNA fragments for various regions of the DE- and DN-cadherin ectodomains were PCR-amplified using the primers and templates described listed in Table S1 and inserted into the *Not*I site of pAcHis-EGFP and/or pAc5.1/V5-His A. To generate the DNA constructs described in Fig. S2, the In-Fusion HD Cloning Kit was used after DNA fragments were amplified by PCR with the primers and templates listed in Table S1 and pUAST (Brand and Perrimon, 1993) was digested with the restriction enzyme *Not*I. Expression of the expected products from the constructed plasmids was assessed by transient transfection and western blotting.

### Cell culture

S2 cells, which were originally obtained from the *Drosophila* community 30 years ago (Schneider, 1972), were cultured at 25°C in Schneider's medium (Thermo Fisher Scientific) supplemented with 10% heat-inactivated fetal bovine serum, unless otherwise indicated. Before experimentation, we confirmed by western blotting analysis that S2 cells used expressed no detectable levels of DE- or DN-cadherin. All transfections were performed using *Trans*IT^®^-Insect Transfection Reagent (MIR 6100; Mirus Bio, Madison, WI, USA) according to manufacturer's instructions.

### Protein expression analysis

Cell lysates, culture supernatants and purified proteins were separated by SDS-PAGE using 7.5% or 6% gels, followed by Coomassie Brilliant Blue staining or western blotting. The antibodies used for western blotting were as follows: rat anti-DN-cadherin antibody (DN-Ex#8; 1:1000; Iwai et al., 1997), rat anti-DE-cadherin antibody (DCAD2; 1:100; Oda et al., 1994), mouse anti-V5 antibody (#46-0705; 1:1000; Thermo Fisher Scientific), mouse anti-GFP antibody (#632375; 1:1000; Takara Bio, Shiga, Japan), and horseradish peroxidase (HRP)-conjugated anti-rat (NA935V) and anti-mouse (NA931V) IgG antibodies (1:1000; GE Healthcare, Chicago, IL, USA). ECL western blotting detection reagents (GE Healthcare) were used for signal detection. Some western blots were re-probed according to the manufacturer's instructions.

The DNEXf-G products were affinity-purified from ∼27 ml conditioned medium of S2 cells transiently transfected with pAcHis-DNEXf-G as described below for AFM protein preparation. Polypeptides eluted with the V5 tag peptide were concentrated using MINICENT-30 (TOSOH, Tokyo, Japan), separated by SDS-PAGE and blotted onto PVDF membrane. The blot was briefly stained with 0.1% Coomassie Brilliant Blue R-250, washed with 50% methanol, and rinsed with water. Visible 250 kDa and 125 kDa protein signals (Fig. S1B) were excised and subjected to N-terminal peptide sequencing (Nippi, Tokyo, Japan).

### Bead aggregation assay

S2 cells were seeded at a density of ∼5×10^5^/ml in 5 ml medium in 60-mm dishes (150462; Thermo Fisher Scientific) ∼7 h before transfection; 5 µg of plasmid DNA was used for each transfection. After 4 days of incubation, bead aggregation assays were performed using conditioned medium at room temperature. Each medium was centrifuged at 18,000 ***g*** to collect the supernatant. Anti-His-tag magnetic beads (D291-11, 5 µl; Medical & Biological Laboratories, Nagoya, Japan) was added to 500 µl of each supernatant in a 1.5 ml tube and immediately mixed. The beads suspensions were transferred to a four-well plate (179820; Thermo Fisher Scientific), followed by 10 min of rotation (32 mm diameter) at 150 rpm on a horizontal shaker (NR-3; TAITEC, Saitama, Japan), after which the four-well plate was kept still for 20 min so that the beads could settle onto the bottom of the plate. The plate was then slowly relocated onto the stage of a nearby inverted microscope (Eclipse Ts2; Nikon, Tokyo, Japan). Bead distribution was photographed to obtain one image (2048×2880 pixels; pixel size, 0.943 µm) for each well using a 4× objective lens and a digital color camera (DS-Fi3; Nikon). Before completing image acquisition for multiple samples in the plate, bead aggregates were prevented from growing by keeping them still. The images were systematically trimmed to 2000×2000 pixels and processed using the ‘Sharpen’ and ‘Enhance Contrast’ functions with the same settings and analyzed using the ‘Analyze Particles’ function in the ImageJ 1.51d software (National Institutes of Health, Bethesda, MD, USA; Schindelin et al., 2012) to measure the areas of bead aggregates. The aggregate sizes were categorized into five successive ranges and the sum of areas of aggregates in each range was calculated. At least three independent transfections were performed to measure the ability of each construct to induce bead aggregates. For bead aggregation assays using purified cadherin ectodomain fragments, the proteins were diluted in 500 µl of HCM buffer (20 mM HEPES, 4 mM CaCl_2_, and 10 mM MgCl_2_, adjusted with NaOH to pH 7.35) containing 1% bovine serum albumin (BSA, A-2153; Sigma-Aldrich, St Louis, MO, USA), into which 5 µl of anti-His-tag magnetic beads was added, followed by the same procedure as described above.

### Cell aggregation assay

S2 cells were seeded at a density of ∼5×10^5^/ml in 5 ml medium in 60-mm dishes ∼7 h before transfection. A mixture of 4.5 µg pUAST-DNECX-TMCP-G (where X is 8–15; Table S1) and 0.5 µg pWA-GAL4 (a gift from Yasushi Hiromi at National Institute of Genetics, Japan) was used for each transfection. At ∼40 h after transfection, cells were collected from each dish and suspended in 4 ml of medium, where the cell density was ∼1.8×10^6^/ml. Then, 100 µl of each cell suspension was added to 400 µl medium, which was transferred to a well in a 24-well plate. The plate was rotated at 150 rpm on a horizontal shaker for 10 min at room temperature, after which the plate was kept still for 10 min so that the cells could settle onto the bottom of the plate. Cells in the wells were photographed using an inverted fluorescence microscope equipped with DIC optics, a 10× objective lens and a cooled CCD camera (CoolSNAP HQ; Roper Scientific, Tucson, AZ, USA) controlled by MetaMorph ver. 6.1 (Molecular Devices, San Jose, CA, USA). For the mixed cell aggregation assay, a mixture of 2.5 µg pUAST-X (where X is DN-cad, DE-cad, DNEC11-TMCP-G, or DEEC6-TMCP-G), 2.0 µg pUAST-Y (where Y is BFPtag or mKate2) and 0.5 µg pWA-GAL4 was used for each transfection. Transfected cells were collected and suspended in medium as described above, after which 100 µl each of the two different cell suspensions was added to 300 µl medium and transferred to a well, followed by rotation.

### Antiserum production

A total of 40 µg of pAcHis-DNPREC2 (Table S1) was used for transfection to express the 435-amino-acid (aa) region (aa 1–434) of DN-cadherin in S2 cells in 10 ml×4 medium at 25°C. At 4 days after transfection, 35 ml of the cell culture supernatant was collected and concentrated to ∼1.5 ml using an Amicon Ultra-15 Centrifugal Filter Unit, 30 kDa cutoff (Sigma-Aldrich), followed by addition of 6.5 ml of HC buffer (20 mM HEPES pH 7.35 and 4 mM CaCl_2_) and 850 µl anti-V5-tag magnetic beads (M215-11; Medical & Biological Laboratories, Nagoya, Japan). After 1.5 h of vertical rotation at 4°C, the beads were washed with 8 ml HC buffer twice and with 1.4 ml HC buffer once followed by suspension in 850 µl of HC buffer. This bead suspension was divided into eight aliquots, which were stored at −80°C and used as antigens to immunize two mice. Antisera from the mice were confirmed to detect specific products derived from pAcHis-DNPREC2 and pAcHis-DNEC2 (Fig. S1C). Although the antigen included the V5/6×His tag sequence, the obtained antisera did not react to the C-terminal tag portion of the products at detectable levels on western blots (Fig. S1C). One of the antisera was used for western blotting at a dilution of 1:400.

### Protein purification

S2 cells were seeded at a density of ∼5×10^5^/ml in 10 ml medium supplemented with 10% heat-inactivated fetal bovine serum in a 90-mm dish (Sumitomo Bakelite, Tokyo, Japan). Following overnight incubation at 28°C, the cells were transfected with expression plasmids for V5-His-tagged proteins. After 3 days of incubation at 28°C, the conditioned medium containing secreted V5-His-tagged proteins were collected and centrifuged at 400 ***g*** for 5 min at 4°C to obtain 10 ml of supernatants, which were passed through a 0.22-μm pore size filter. To purify V5-His-tagged proteins from the supernatants, the V5-tagged Protein Magnetic Purification Kit (Medical & Biological Laboratories) was used according to manufacturer's instructions. For AFM, the buffer of purified proteins was exchanged for HCM buffer using Micro Bio-Spin^®^ Columns with Bio-Gel^®^ P-30 (Bio-Rad Laboratories, Hercules, CA, USA). Purified proteins were stored at 4°C until use.

### Protein preparation for AFM

A mica substrate with a diameter of 3 mm and a thickness of 0.1 mm (Furuuchi Chemical, Tokyo, Japan) was attached with glue in the center of 15-mm-diameter hydrophilic circles on a glass slide (Matsunami Glass Industries, Osaka, Japan). Purified proteins (2 μl) in solution (HCM buffer) were adsorbed to freshly cleaved mica for 15 min, after which the mica surface was washed twice with 250 μl HCM buffer. After washing, the buffer was replaced with HCM buffer containing 1% glutaraldehyde (GA). The concentration of the proteins used for adsorption was adjusted based on pilot observations. These processes were conducted at room temperature (25°C).

### AFM imaging

AFM imaging of proteins in solution was performed at room temperature (25°C) using a tip-scan type atomic force microscope (BIXAM; Olympus, Tokyo, Japan), as previously described (Suzuki et al., 2013; Nishiguchi et al., 2016). The AFM was set to the phase modulation mode. Customized cantilevers (USC-F0.8-k0.1; Nano World AG, Neuchâtel, Switzerland), with a length of 9 μm, width of 2 μm, thickness of 0.10 μm, and a spring constant of 0.1 N/m, were used for AFM image acquisition. The cantilever had a scanning tip radius of less than 10 nm, a resonant frequency in a solution of 300 kHz. The scanning area for high-resolution images (Fig. 6) was 120×90 nm^2^ or 180×135 nm^2^ (320×240 pixels), and 300×225 nm^2^ or 480×360 nm^2^ (320×240 pixels) for other images (Figs 5 and 7; Figs S3, S4, S6). All AFM images were acquired at a frame rate of 0.1 fps and exported as bmp files. At least two independent protein purifications were performed to examine each molecular type by AFM.

### AFM image processing and analysis

AFM image processing and molecular dimension measurements were performed using the ImageJ 1.53a software. Most AFM images were processed with a median filter (3×3 pixels) and mean filter (3×3 pixels). The background was subtracted to show the height relative to the mica surface and adjusted for contrast. For dimension measurements in Fig. 5, Figs S3 and S5, images with a scanning area of 480×360 nm^2^ (320×240 pixels) were binarized and individual objects were subjected to ellipse fitting (‘Fit Ellipse’ command in ImageJ). For measurement analyses of DNEXf and DEEXf, manually selected objects that showed the typical morphological features were used. To measure the approximate relative volumes of the strand-like and globule-like portions of cadherin ectodomains, images with a scanning area of 120×90 nm^2^ or 180×135 nm^2^ (320×240 pixels) were manually segmented.

The Gabor filter (Gabor, 1946; Daugman, 1987, 1980), which was used for segmentation of the AFM images, is given by:(1)
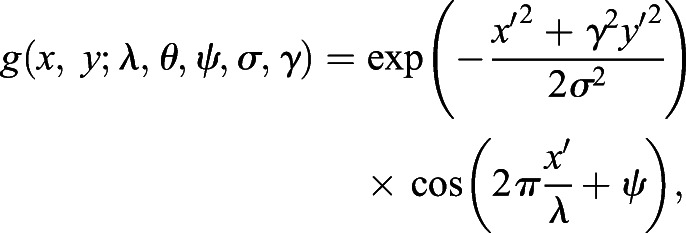
where
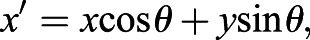
and
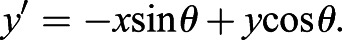
In this equation, we set the parameters as: *λ*=20, *ψ*=0, *σ*=2, *γ*=0.4. When *θ*=0, the filter with a size of 12×12 pixels (Fig. S6B) optimally extracts horizontal straight stripes with a width of 4–6 pixels, which corresponds to EC repeats (∼6–9 nm width via AFM) in low-magnification AFM images (240×240 pixels, 1.5 nm/pixel) (Fig. S6). To enhance the extraction of straight stripes irrespective of the angle, 12 serial Gabor filters (*θ*=0, 15, 30, …, 165 [deg]) were prepared. Each filter was applied to a binary image to obtain the output *h* as follows:(2)
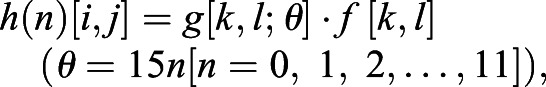
where *i*, *j*, *k*, and *l* are the pixel positions of the image and filter, respectively, and *f* is the binary image. The outputs for the 12 angles were combined to obtain the final Gabor filter output *G* as follows:(3)
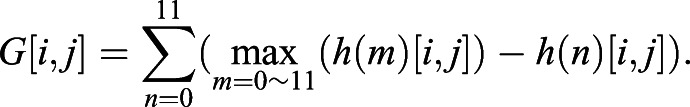
Application of Gabor filter processing to binary images of pseudo molecules demonstrated extraction of stripe features from the pseudo molecules with preferential exclusion of knotted and kinked regions (Fig. S6D–F). The Gabor filter outputs (magenta) were merged with normalized AFM height images (green) in pseudo colors (Fig. 7B). These resultant segmented images helped recognize the morphological features of individual cadherin fragments imaged by AFM, which aided morphological categorization. Gabor filter processing was performed using an in-house program written in C#, which is available upon request.

For comparative morphological characterization of DNEC14, DNEC11, DNEC8, and DNEC5, low-magnification AFM images (240×240 pixels, 1.5 nm/pixel) that had molecular objects with clear outlines at appropriate densities were selected and analyzed. For systematic object selection, the area of all individual objects was measured after the images were exceptionally processed with a median filter of 5×5 pixels and binarized. Of these objects, those with an area ranging within the mean±1 s.d. were considered for lateral dimension measurement, segmentation and categorization.

## Supplementary Material

Supplementary information

Reviewer comments
