# Supplementary material for: Structural variability and dynamics in the ectodomain of an ancestral-type classical cadherin revealed by AFM imaging
Source: J Cell Sci. 2021 Jul 22;134(14):jcs258388. doi: 10.1242/jcs.258388 (PMC8325961; doi:10.1242/jcs.258388)
Supplement: Supplementary information [file joces-134-258388-s1.pdf]

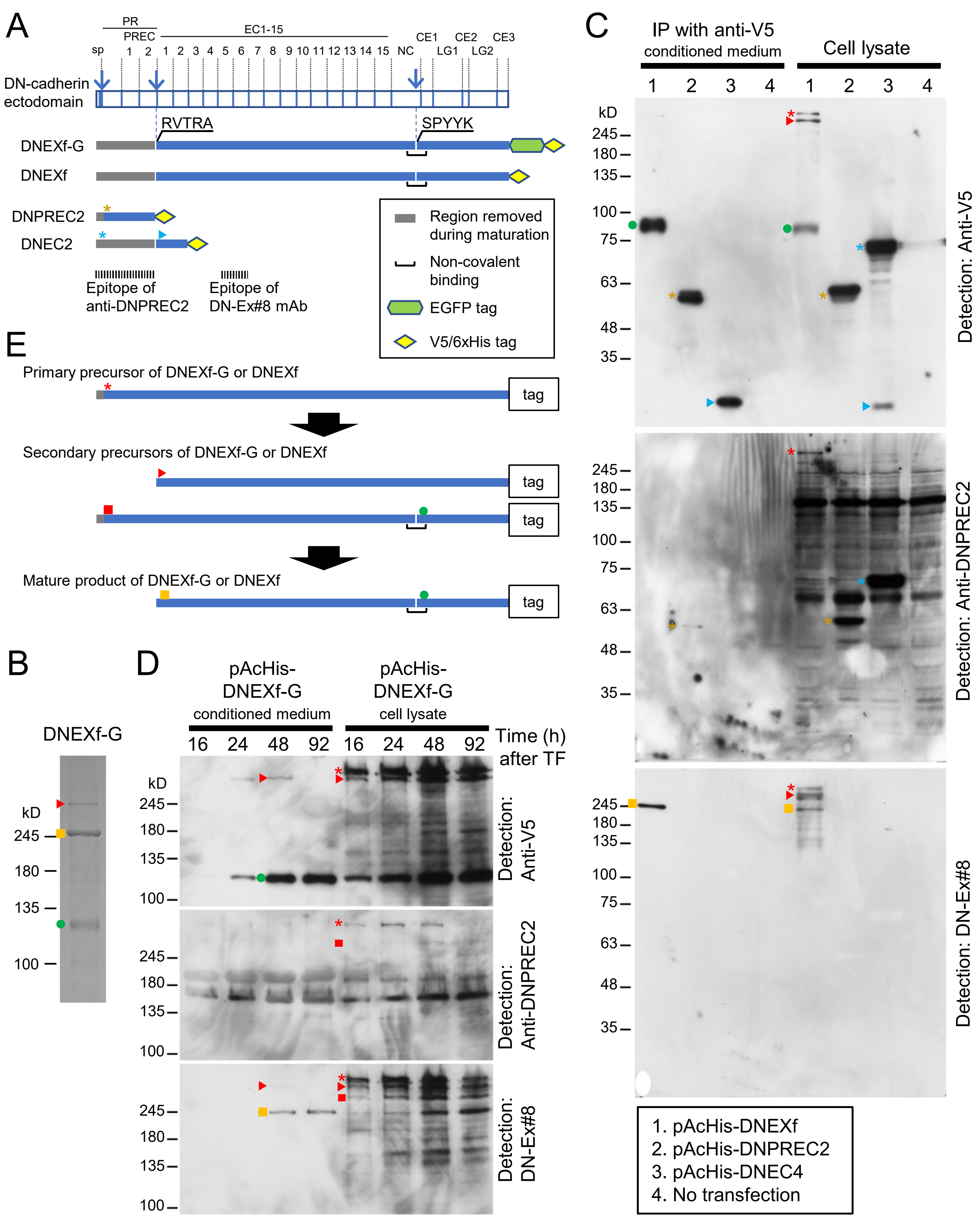

Fig. S1. Characterization of DN-cadherin ectodomain processing.

(A) Schematic representation of four DN-cadherin ectodomain constructs used for investigating DN-cadherin ectodomain processing. The determined N-terminal five residues of the membrane-distal and -proximal polypeptides are indicated. (B) CBB staining of purified membrane-distal and -proximal polypeptides of DNEXf-G blotted on polyvinylidene difluoride membrane. A precursor product is also visible. (C) Western

Journal of Cell Science • Supplementary information

blot detection of products containing the first two EC repeats. Four populations of S2 cells were prepared, which were transfected with the plasmids pAcHis-DNEXf, pAcHis-DNPREC2, and pAcHis-DNEC2 and not transfected. Immunoprecipitation from conditioned media of these cells with ant-V5 antibody was performed. The cell lysates and immunoprecipitates were examined by western blotting with the antibodies indicated. (D) Time-course analysis of the products of DNEXf-G in conditioned media and cell lysates at four different time points after transfection (16, 24, 48, and 92 h). (E) Schematic representation of possible precursor products from the full-length DN-cadherin ectodomain constructs. Polypeptides of different sizes are marked with a red asterisk, red triangle, red square, green circle, and yellow square. In the blots shown in B, C, and D, the relevant polypeptide signals are indicated using the same marks.

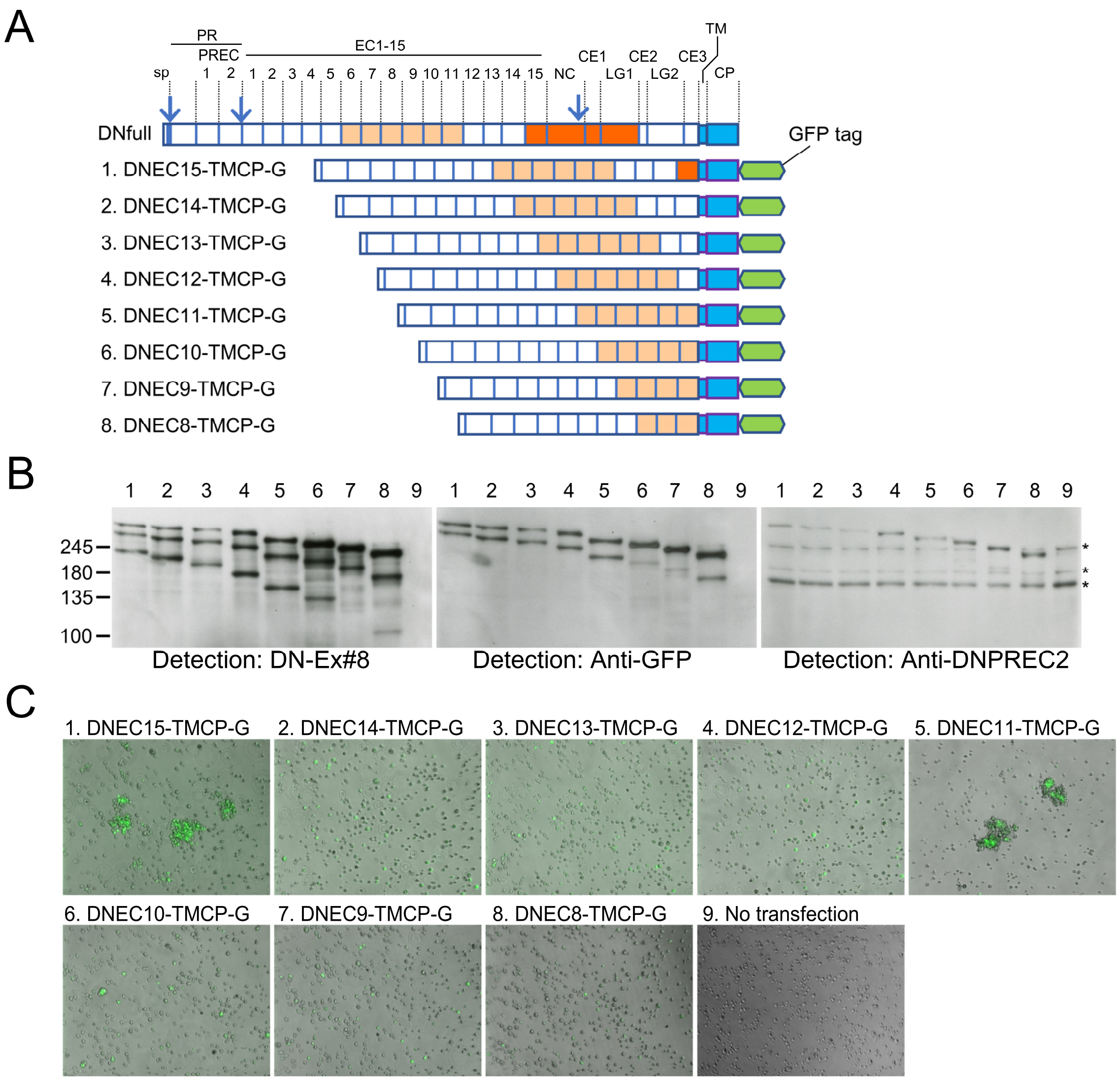

Fig. S2. A series of DN-cadherin ectodomain deletion constructs for testing cell-cell binding capability.

(A) Schematic representation of a series of DN-cadherin ectodomain deletions fused with DE-cadherin TM and CP as well as a GFP tag. These chimeric cadherins, numbered from 1 to 8, were expressed in S2 cells by transient transfection. (B) Western blotting of the expression of the constructed chimeric cadherins in S2 cells. In control experiments (lane 9), S2 cells that were not transfected were used. Asterisks indicate three non-specific protein signals. (C) Cell aggregation assay using the S2 cells. Differential interference contrast and GFP fluorescence images are merged. Two of the eight deletion constructs (numbers 1 and 5) were capable of inducing cell aggregates.

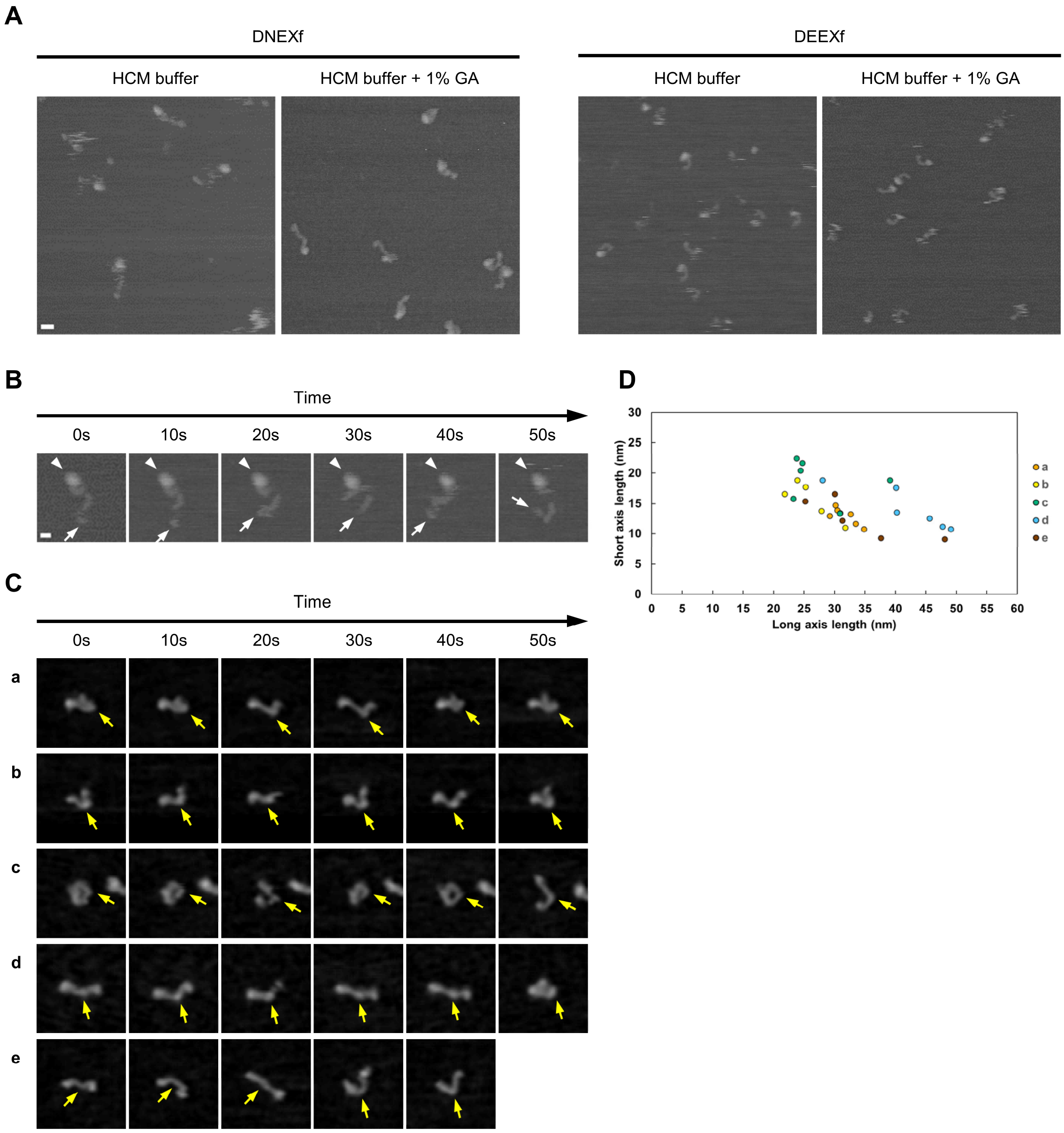

Fig. S3. Raw AFM images of DN- and DE-cadherin ectodomains.

(A) Raw AFM images of DNEXf molecules acquired in HCM buffer or buffer containing 1% GA (HCM-GA buffer). (B) Sequential scanning images of a single DNEXf molecule in HCM buffer. White arrows indicate the strand-like portion, and white arrowheads the globule-like portion. See also Movie S1. (C) Sequential scanning images of five additional examples of DNEC14 molecules acquired in HCM-GA buffer. Yellow arrows point to the sites of flexible bending in the molecules. Scale bars, 20 nm in A; 10 nm in B and C. (D) The dimension values at different time points in C and individual molecules are indicated by the alphabet used in C.

Journal of Cell Science • Supplementary information

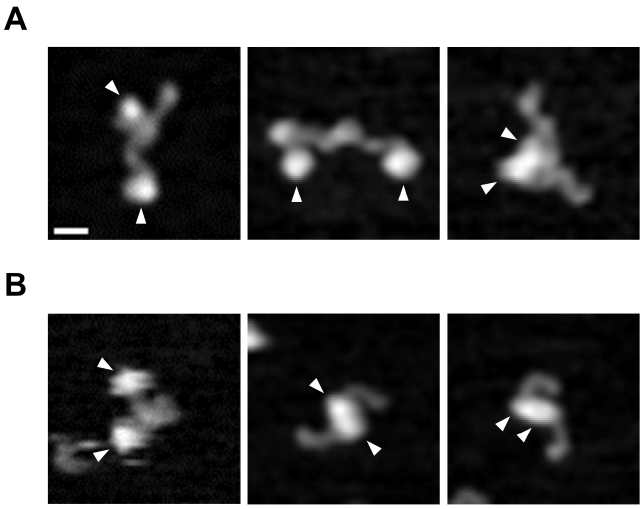

Fig. S4. Rare detection of dimer-like pairs for DNEXf and DEEXf.

(A, B) Examples of dimer-like pairs for DNEXf (A) and DEEXf (B). Arrowheads indicate the globule-like portion of each molecule. Scale bar, 10 nm. All images are displayed at the same scale.

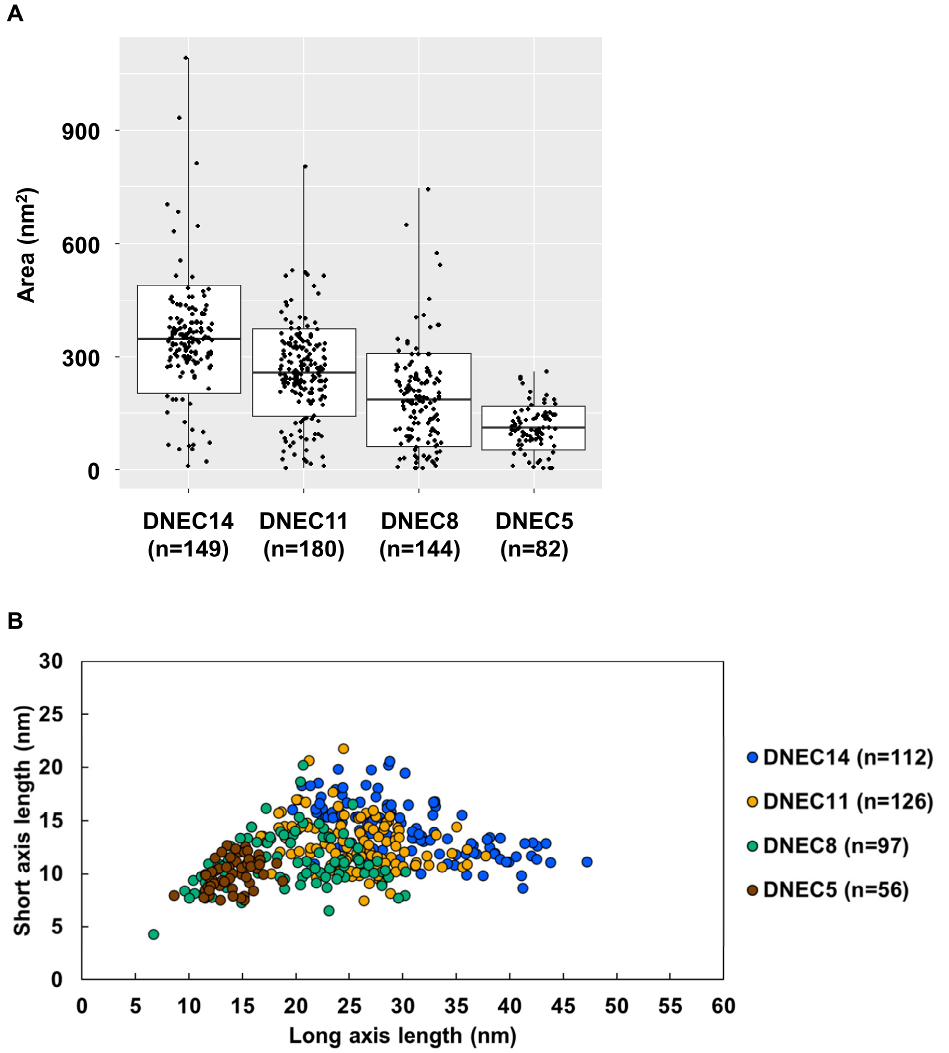

Fig. S5. Dimensional variations of AFM-imaged DNEC14, DNEC11, DNEC8 and DNEC5 molecules.

(A) Scatter plots showing the area of all objects obtained from AFM images for DNEC14, DNEC11, DNEC8, and DNEC5 each. The boxes and bars indicate the maximum, the mean + SD, the mean, the mean - SD, and the minimum. Objects that have an area ranging within the mean ± SD were selected for the following analyses. (B) Scatter plots showing the short- and long-axis lengths of the selected individual objects from AFM images for DNEC14, DNEC11, DNEC8, and DNEC5 each. The length values were obtained by ellipse fitting.

Journal of Cell Science • Supplementary information

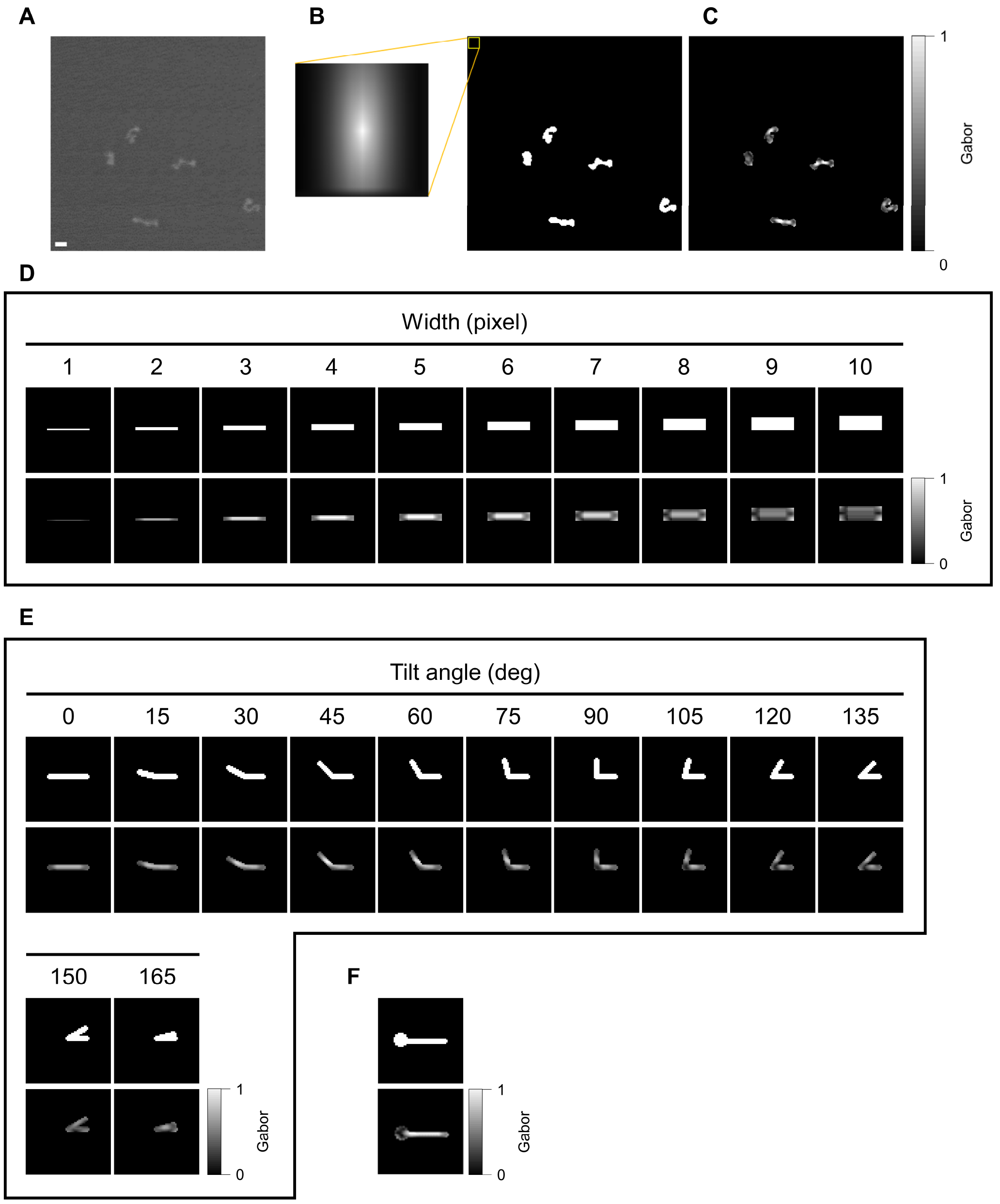

Fig. S6. Demonstration of the Gabor filter processing applied for AFM-imaged EC repeats.

(A–C) A representative AFM raw image for DNEC14 (A) was binarized (B) and then processed by the Gabor filter with a size of 12 x 12 pixels (yellow box in B). The output image with normalized intensities is shown in C. Scale bar, 20 nm. (D, E) Evaluation of width (D) and bend angle (E) effects using binary pattern samples (upper images). The output images are displayed in the lower layer. (F) Ability of the Gabor filter processing to negatively detect a knot-like region in a binary pattern sample (upper image). The output image is displayed in the lower position.

Journal of Cell Science • Supplementary information

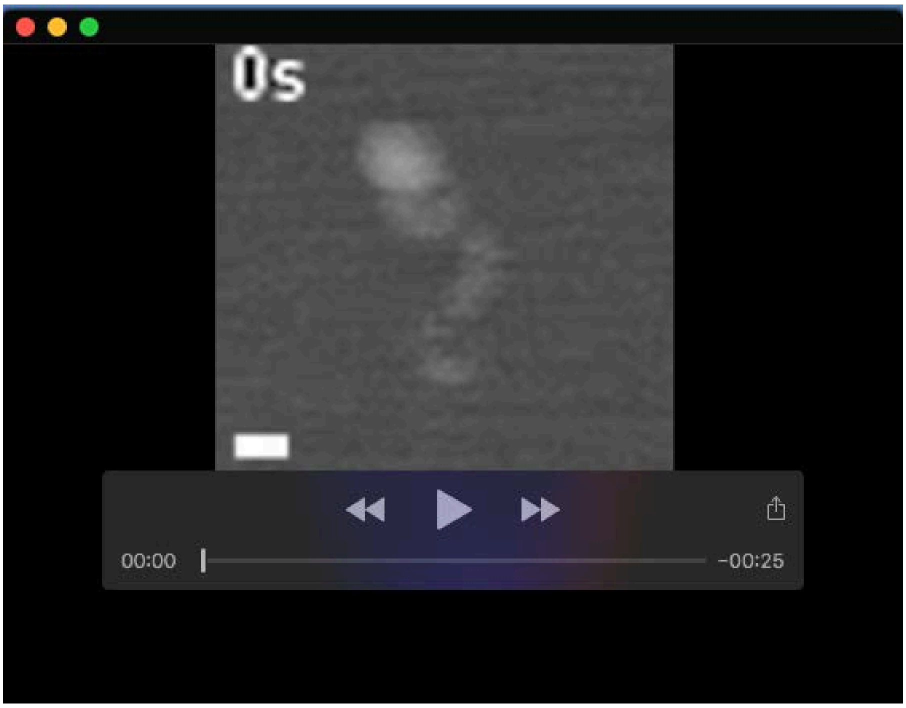

Movie 1. Sequential scanning images of a single DNEXf molecule in HCM buffer.
This movie is related to Fig. S3B. Scale bar, 10 nm.

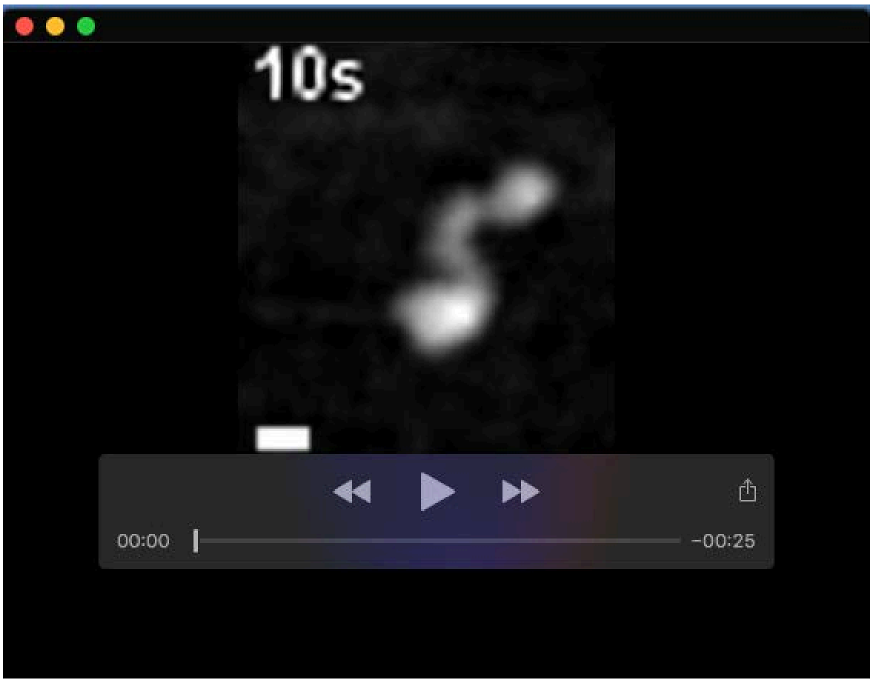

Movie 2. Sequential scanning images of a single DNEXf molecule in HCM-GA buffer.
This movie is related to Fig. 5D. Scale bar, 10 nm.

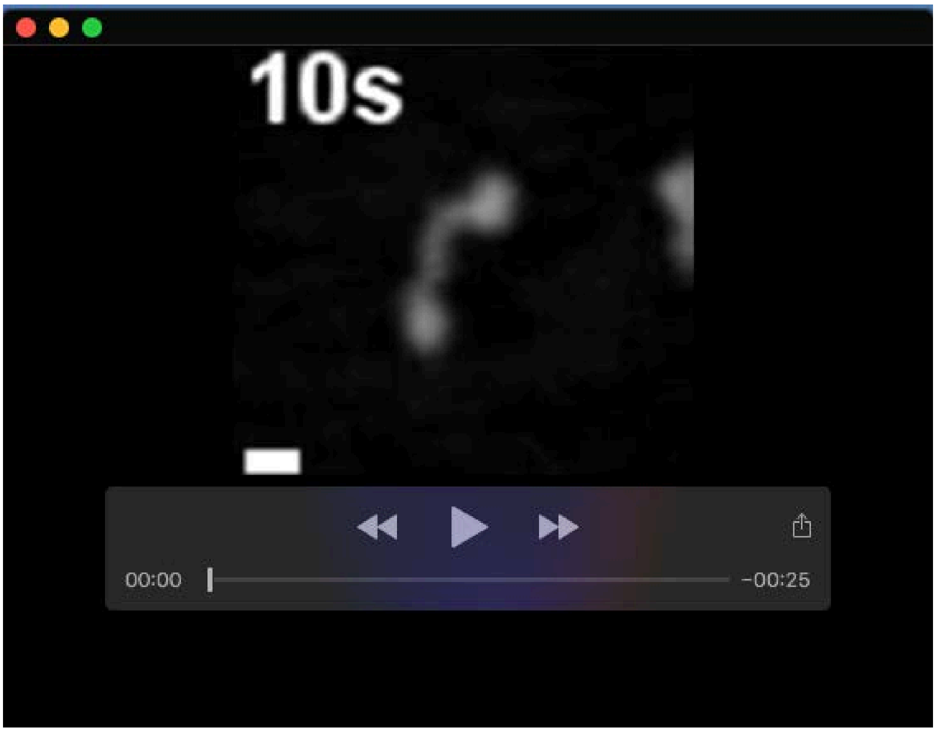

Movie 3. Sequential scanning images of a single DNEC14 molecule in HCM-GA buffer.

This movie is related to Fig. 5I. Scale bar, 10 nm.

Table S1. Primers used for DNA construction.

Construct	1: PCR template for insert preparation<br>2: Vector (insertion site)	Primers used (Recognition sequences for restriction enzymes used are indicated with underlining)<br>F: Forward primer<br>R: Reverse primer
pUP-DEEXf-G	1: pBS-DECH[#1]<br>2: pUP-Arm-EGFP[#2] (NcoI-NheI)	F: 5'-CATGTCATGAGCACCAGTGTCCAGCGAATG-3'<br>R: 5'-CCTAGCTAGCCGACTGCTGGCAACCGCTGTC-3'
pAcHis-DEEXf-G	1: pUP-DEEXf-G<br>2: pAc5.1/V5-His A (EcoRI-XbaI)	F: 5'-GGGAATTCATGTCCACCAGTGTCCAGCGAATG-3'<br>R: 5'-GCTCTAGACTTGTACAGCTCGTCCAT-3'
pAcHis-DEEXf	1: pBS-DECH<br>2: pAc5.1/V5-His A (EcoRI-XbaI)	F: 5'-GGGAATTCATGTCCACCAGTGTCCAGCGAATG-3'<br>R: 5'-GCTCTAGACGACTGCTGGCAACCGCTGTC-3'
pAcHis-EGFP	1: pAcHis-DEEC6-EGFP[#3]<br>2: pAc5.1/V5-His A (XhoI-PmeI)	F: 5'-GGGCTCGAGGCTAGCAAGGGCGAGGAGCTG-3'<br>R: 5'-TAGAAGGCACAGTCGAGG-3'
pAcHis-DNEXf-G	1: pUAST-DN-cad[#4]<br>2: pAcHis-EGFP (NotI)	F: 5'-ATTTGCGGCCGCATGGCGGCACGACGTTGC-3'<br>R: 5'-TAAAGCGGCCGCCCTTTTAAGGTCTGACCCTCCTG-3'
pAcHis-DNEXf	1: pUAST-DN-cad<br>2: pAc5.1/V5-His A (NotI)	F: 5'-ATTTGCGGCCGCATGGCGGCACGACGTTGC-3'<br>R: 5'-TAAAGCGGCCGCCCTTTTAAGGTCTGACCCTCCTG-3'
pAcHis-DEEXd-G	1: pUAST-dCR3h[#1]<br>2: pAcHis-EGFP (EcoRI-NheI)	F: 5'-GGGAATTCATGTCCACCAGTGTCCAGCGAATG-3'<br>R: 5'-GCTCTAGACGACTGCTGGCAACCGCTGTC-3'
pAcHis-DNLG1-G	1: pUAST-DN-cad<br>2: pAcHis-EGFP (NotI)	F: 5'-ATTTGCGGCCGCATGGCGGCACGACGTTGC-3'<br>R:<br>5'-TAAAGCGGCCGCCCGGTTTGAGGACAACCCGCGAC-3'
pAcHis-DNNC-G	1: pUAST-DN-cad<br>2: pAcHis-EGFP (NotI)	5'-ATTTGCGGCCGCATGGCGGCACGACGTTGC-3'<br>5'-TAAAGCGGCCGCCCCTCATCGATGCCCACCATGGT-3'
pAcHis-DNEC17-G	1: pUAST-DN-cad<br>2: pAcHis-EGFP (NotI)	F: 5'-ATTTGCGGCCGCATGGCGGCACGACGTTGC-3'<br>R: 5'-TAAAGCGGCCGCCCTGGCTGCATTTTGTTGTCGTT-3'
pAcHis-DNEC16-G	1: pUAST-DN-cad<br>2: pAcHis-EGFP (NotI)	F: 5'-ATTTGCGGCCGCATGGCGGCACGACGTTGC-3'<br>R: 5'-TAAAGCGGCCGCCCGAATTTGGGAGCATTGTC-3'
pAcHis-DNEC15-G	1: pUAST-DN-cad<br>2: pAcHis-EGFP (NotI)	F: 5'-ATTTGCGGCCGCATGGCGGCACGACGTTGC-3'<br>R: 5'-TAAAGCGGCCGCCCGAAATGCGGCTTATTGTCGTT-3'
pAcHis-DNEC14-G	1: pUAST-DN-cad<br>2: pAcHis-EGFP (NotI)	F: 5'-ATTTGCGGCCGCATGGCGGCACGACGTTGC-3'<br>R: 5'-TAAAGCGGCCGCCCAAACTGCGGCGGCATATCGTT-3'

Journal of Cell Science • Supplementary information

pAcHis-DNEC13-G	1: pUAST-DN-cad<br>2: pAcHis-EGFP (NotI)	F: 5'-ATTTGCGGCCGCATGGCGGCACGACGTTGC-3'<br>R: 5'-TAAAGCGGCCGCCCGAAAATGGGGGCATTATC-3'
pAcHis-DNEC12-G	1: pUAST-DN-cad<br>2: pAcHis-EGFP (NotI)	F: 5'-ATTTGCGGCCGCATGGCGGCACGACGTTGC-3'<br>R: 5'-TAAAGCGGCCGCCCAAAAACGGGGGGATTATCGTT-3'
pAcHis-DNEC11-G	1: pUAST-DN-cad<br>2: pAcHis-EGFP (NotI)	F: 5'-ATTTGCGGCCGCATGGCGGCACGACGTTGC-3'<br>R: 5'-TAAAGCGGCCGCCCGAAATAAGGCGGGTTATCGTT-3'
pAcHis-DNEC10-G	1: pUAST-DN-cad<br>2: pAcHis-EGFP (NotI)	F: 5'-ATTTGCGGCCGCATGGCGGCACGACGTTGC-3'<br>R: 5'-TAAAGCGGCCGCCCGAATAAAGGTATTTCATCATT-3'
pAcHis_DNEC9-G	1: pUAST-DN-cad<br>2: pAcHis-EGFP (NotI)	F: 5'-ATTTGCGGCCGCATGGCGGCACGACGTTGC-3'<br>R: 5'-TAAAGCGGCCGCCCCAGACGGGCGGATTATTTGC-3'
pAcHis_DNEC8-G	1: pUAST-DN-cad<br>2: pAcHis-EGFP (NotI)	F: 5'-ATTTGCGGCCGCATGGCGGCACGACGTTGC-3'<br>R: 5'-TAAAGCGGCCGCCCGAACAGTGGCGGATTATCGTT-3'
pAcHis-DNEC7-G	1: pUAST-DN-cad<br>2: pAcHis-EGFP (NotI)	F: 5'-ATTTGCGGCCGCATGGCGGCACGACGTTGC-3'<br>R: 5'-TAAAGCGGCCGCCCGAACACTGGCTTATTATCGTT-3'
pAcHis_DNEC6-G	1: pUAST-DN-cad<br>2: pAcHis-EGFP (NotI)	F: 5'-ATTTGCGGCCGCATGGCGGCACGACGTTGC-3'<br>R: 5'-TAAAGCGGCCGCCCGAACTTGGGCTCCTCATCGTT-3'
pAcHis_DNEC5-G	1: pUAST-DN-cad<br>2: pAcHis-EGFP (NotI)	F: 5'-ATTTGCGGCCGCATGGCGGCACGACGTTGC-3'<br>R: 5'-TAAAGCGGCCGCCCGAACTTCGGCGCGTTATCATT-3'
pAcHis-DNEC16	1: pUAST-DN-cad<br>2: pAc5.1/V5-His A (NotI)	5'-ATTTGCGGCCGCATGGCGGCACGACGTTGC-3'<br>5'-TAAAGCGGCCGCCCGAATTTGGGAGCATTGTC-3'
pAcHis-DNEC13	1: pUAST-DN-cad<br>2: pAc5.1/V5-His A (NotI)	F: 5'-ATTTGCGGCCGCATGGCGGCACGACGTTGC-3'<br>R: 5'-TAAAGCGGCCGCCCGAAAATGGGGGCATTATC-3'
pAcHis-DNEC10	1: pUAST-DN-cad<br>2: pAc5.1/V5-His A (NotI)	F: 5'-ATTTGCGGCCGCATGGCGGCACGACGTTGC-3'<br>R: 5'-TAAAGCGGCCGCCCGAATAAAGGTATTTCATCATT-3'
pAcHis-DNEC7	1: pUAST-DN-cad<br>2: pAc5.1/V5-His A (NotI)	5'-ATTTGCGGCCGCATGGCGGCACGACGTTGC-3'<br>5'-TAAAGCGGCCGCCCGAACACTGGCTTATTATCGTT-3'
pAcHis-DNEC4	1: pUAST-DN-cad<br>2: pAc5.1/V5-His A (NotI)	F: 5'-ATTTGCGGCCGCATGGCGGCACGACGTTGC-3'<br>R: 5'-TAAAGCGGCCGCCCTCTTTTCTCTCGATGATG-3'
pAcHis-DNPREC2	1: pUAST-DN-cad<br>2: pAc5.1/V5-His A (NotI)	F: 5'-ATTTGCGGCCGCATGGCGGCACGACGTTGC-3'<br>R: 5'-TAAAGCGGCCGCCCGAACTGCGGTGCTCGTTTGCC-3'
pAcHis-DENC-G	1: pBS-DECH<br>2: pAcHis-EGFP (NotI)	F: 5'-ATTTGCGGCCGCATGTCCACCAGTGTCCAG-3'<br>R: 5'-TAAAGCGGCCGCCCCTCGTCGATGTTCACCATCAG-3'

Journal of Cell Science • Supplementary information

pAcHis-DEEC7-G	1: pBS-DECH<br>2: pAcHis-EGFP (NotI)	F: 5'-ATTTGCGGCCGCATGTCCACCAGTGTCCAG-3'<br>R: 5'-TAAAGCGGCCGCCCTTCGCTCATGGCATTGTCGTT-3'
pAcHis-DEEC6-G	1: pBS-DECH<br>2: pAcHis-EGFP (NotI)	F: 5'-ATTTGCGGCCGCATGTCCACCAGTGTCCAG-3'<br>R: 5'-TAAAGCGGCCGCCCCAGGAACGGAGCATTGTCGTT-3'
pAcHis-DEEC5-G	1: pBS-DECH<br>2: pAcHis-EGFP (NotI)	F: 5'-ATTTGCGGCCGCATGTCCACCAGTGTCCAG-3'<br>R: 5'-TAAAGCGGCCGCCCGAACACCGGTGGGTTGTCGTT-3'
pAcHis-DEEC4-G	1: pBS-DECH<br>2: pAcHis-EGFP (NotI)	F: 5'-ATTTGCGGCCGCATGTCCACCAGTGTCCAG-3'<br>R: 5'-TAAAGCGGCCGCCCGAAGTGCGGCTTGTGGTCATT-3'
pAcHis-DEEC3-G	1: pBS-DECH<br>2: pAcHis-EGFP (NotI)	F: 5'-ATTTGCGGCCGCATGTCCACCAGTGTCCAG-3'<br>R: 5'-TAAAGCGGCCGCCCGTAGTAGGGGATGTTATCGTT-3'
Construct	1: PCR templates for insert preparation<br>t1, template 1;<br>t2, template 2<br>2: Vector (insertion site)	Primers used (Sequences for the homologous ends required for In-Fusion cloning are indicated with underlining)<br>t1F: Forward primer<br>t1R: Reverse primer<br>t2F: Forward primer<br>t2R: Reverse primer
pUAST-DNEC15-TMCP-G	1:<br>t1, pUAST-DN-cad<br>t2, pUAST-DEFL[#1]<br>2: pUAST (NotI)	t1F:<br>5'-AACAGATCTGCGGCCGCATGGCGGCACGACGTTGC-3'<br>t1R: 5'-GAACGCAACGGCCACTGGCTGCATTTTGTTGTC-3'<br>t2F: 5'-GTGGCCGTTGCGTTCAGTTTTGGA-3'<br>t2R: 5'-CCTCGAGCCGCGGCCGCGGCTGGCGAAGATTC-3'
pUAST-DNEC14-TMCP-G	1:<br>t1, pUAST-DN-cad<br>t2, pUAST-DEFL<br>2: pUAST (NotI)	t1F:<br>5'-AACAGATCTGCGGCCGCATGGCGGCACGACGTTGC-3'<br>t1R: 5'-GAACGCAACGGCCACGAATTTGGGAGCATTGTC-3'<br>t2F: 5'-GTGGCCGTTGCGTTCAGTTTTGGA-3'<br>t2R: 5'-CCTCGAGCCGCGGCCGCGGCTGGCGAAGATTC-3'
pUAST-DNEC13-TMCP-G	1:<br>t1, pUAST-DN-cad<br>t2, pUAST-DEFL<br>2: pUAST (NotI)	t1F:<br>5'-AACAGATCTGCGGCCGCATGGCGGCACGACGTTGC-3'<br>t1R: 5'-GAACGCAACGGCCACGAAATGCGGCTTATTGTC-3'<br>t2F: 5'-GTGGCCGTTGCGTTCAGTTTTGGA-3'<br>t2R: 5'-CCTCGAGCCGCGGCCGCGGCTGGCGAAGATTC-3'

Journal of Cell Science • Supplementary information

pUAST-DNEC12-TMCP-G	1:<br>t1, pUAST-DN-cad<br>t2, pUAST-DEFL<br>2: pUAST (NotI)	t1F:<br>5'-AACAGATCTGCGGCCGCATGGCGGCACGACGTTGC-3'<br>t1R: 5'-GAACGCAACGGCCACAAACTGCGGCGGCATATC-3'<br>t2F: 5'-GTGGCCGTTGCGTTCAGTTTTGGA-3'<br>t2R: 5'-CCTCGAGCCGCGGCCGCGGCTGGCGAAGATTC-3'
pUAST-DNEC11-TMCP-G	1:<br>t1, pUAST-DN-cad<br>t2, pUAST-DEFL<br>2: pUAST (NotI)	t1F:<br>5'-AACAGATCTGCGGCCGCATGGCGGCACGACGTTGC-3'<br>t1R: 5'-GAACGCAACGGCCACGAAAATGGGGGCATTATC-3'<br>t2F: 5'-GTGGCCGTTGCGTTCAGTTTTGGA-3'<br>t2R: 5'-CCTCGAGCCGCGGCCGCGGCTGGCGAAGATTC-3'
pUAST-DNEC10-TMCP-G	1:<br>t1, pUAST-DN-cad<br>t2, pUAST-DEFL<br>2: pUAST (NotI)	t1F:<br>5'-AACAGATCTGCGGCCGCATGGCGGCACGACGTTGC-3'<br>t1R: 5'-GAACGCAACGGCCACAAAAACGGGGGGATTATC-3'<br>t2F: 5'-GTGGCCGTTGCGTTCAGTTTTGGA-3'<br>t2R: 5'-CCTCGAGCCGCGGCCGCGGCTGGCGAAGATTC-3'
pUAST-DNEC9-TMCP-G	1:<br>t1, pUAST-DN-cad<br>t2, pUAST-DEFL<br>2: pUAST (NotI)	t1F:<br>5'-AACAGATCTGCGGCCGCATGGCGGCACGACGTTGC-3'<br>t1R: 5'-GAACGCAACGGCCACGAAATAAGGCGGGTTATC-3'<br>t2F: 5'-GTGGCCGTTGCGTTCAGTTTTGGA-3'<br>t2R: 5'-CCTCGAGCCGCGGCCGCGGCTGGCGAAGATTC-3'
pUAST-DNEC8-TMCP-G	1:<br>t1, pUAST-DN-cad<br>t2, pUAST-DEFL<br>2: pUAST (NotI)	t1F:<br>5'-AACAGATCTGCGGCCGCATGGCGGCACGACGTTGC-3'<br>t1R: 5'-GAACGCAACGGCCACGAATAAAGGTATTTCATC-3'<br>t2F: 5'-GTGGCCGTTGCGTTCAGTTTTGGA-3'<br>t2R: 5'-CCTCGAGCCGCGGCCGCGGCTGGCGAAGATTC-3'

References

[#1] **Oda, H. and Tsukita, S.** (1999). Nonchordate classic cadherins have a structurally and functionally unique domain that is absent from chordate classic cadherins. *Dev. Biol.* **216**, 406-422.

[#2] **Oda, H., Wada, H., Tagawa, K., Akiyama-Oda, Y., Satoh, N., Humphreys, T., Zhang, S. and Tsukita, S.** (2002). A novel amphioxus cadherin that localizes to

epithelial adherens junctions has an unusual domain organization with implications for chordate phylogeny. *Evol. Dev.* **4**, 426-434.

#3 **Nishiguchi, S., Yagi, A., Sakai, N. and Oda, H.** (2016). Divergence of structural strategies for homophilic E-cadherin binding among bilaterians. *J. Cell Sci.* **129**, 3309-3319.

#4 **Iwai, Y., Usui, T., Hirano, S., Steward, R., Takeichi, M. and Uemura, T.** (1997). Axon patterning requires DN-cadherin, a novel neuronal adhesion receptor, in the Drosophila embryonic CNS. *Neuron* **19**, 77-89.
