# Supplementary material for: Structural variability and dynamics in the ectodomain of an ancestral-type classical cadherin revealed by AFM imaging
Source: J Cell Sci. 2021 Jul 22;134(14):jcs258388. doi: 10.1242/jcs.258388 (PMC8325961; doi:10.1242/jcs.258388)
Supplement: Reviewer comments [file joces-134-258388_review_history.pdf]

Structural variability and dynamics in the ectodomain of an ancestral-type classical cadherin revealed by AFM imaging

Shigetaka Nishiguchi and Hiroki Oda
DOI: 10.1242/jcs.258388

Editor: Kathleen Green

Review timeline

Original submission:	8 January 2021
Editorial decision:	3 March 2021
First revision received:	30 April 2021
Editorial decision:	9 June 2021
Second revision received:	13 June 2021
Accepted:	15 June 2021

Original submission

First decision letter

MS ID#: JOCES/2021/258388

MS TITLE: Structural variability and dynamics in the ectodomain of an ancestral-type classical cadherin revealed by AFM imaging

AUTHORS: Shigetaka Nishiguchi and Hiroki Oda
ARTICLE TYPE: Research Article

We have now reached a decision on the above manuscript.

To see the reviewers' reports and a copy of this decision letter, please go to: https://submit-jcs.biologists.org and click on the 'Manuscripts with Decisions' queue in the Author Area. (Corresponding author only has access to reviews.)

As you will see, the reviewers differ in their levels of enthusiasm and extent of issues raised, but overall their concerns prevent me from accepting the paper at this stage. The first reviewer states that the results are not surprising and therefore don't really advance our understanding of cadherin structure/dynamics associations in general. Another reviewers is enthusiastic about the appropriateness of the work for JCS, and raised just a few minor issues. The third feels the work is important, but has detailed technical concerns that would need to be addressed.

If you think that you can deal satisfactorily with the criticisms on revision, particularly those of the second two referees, I would be amenable to see a revised manuscript. Also please do the best you can to argue for the importance/novelty of the work in response to the first referee. We would return the revised paper to the reviewers for their feedback.

We are aware that you may be experiencing disruption to the normal running of your lab that makes experimental revisions challenging. If it would be helpful, we encourage you to contact us to discuss your revision in greater detail. Please send us a point-by-point response indicating where you are able to address concerns raised (either experimentally or by changes to the text) and where you will not be able to do so within the normal timeframe of a revision. We will then

© 2021. Published by The Company of Biologists under the terms of the Creative Commons Attribution License (https://creativecommons.org/licenses/by/4.0/).

provide further guidance. Please also note that we are happy to extend revision timeframes as necessary.

Please ensure that you clearly highlight all changes made in the revised manuscript. Please avoid using 'Tracked changes' in Word files as these are lost in PDF conversion.

I should be grateful if you would also provide a point-by-point response detailing how you have dealt with the points raised by the reviewers in the 'Response to Reviewers' box. Please attend to all of the reviewers' comments. If you do not agree with any of their criticisms or suggestions please explain clearly why this is so.

Reviewer 1

Advance summary and potential significance to field

This manuscript analyzed drosophila type III and typeIVa caderins, DN- and DE-cadherins respectively. A number of deletion mutants were made, giving information of precursor processing of cadherins. The mutants were further used for bead aggregation assays and HS-AFM imaging. Extracellular cadherin domain repeats were highly variable, flexible in morphology with several bendable sites. These are basic features of DN- and DE-
cadherins.

Comments for the author

This paper has shown several new findings on the Drosophila cadherins. The authors however, did not address the mechanism of structure-function relationship of cadherin extracellular regions. Most of the data are expected or too descriptive. Our understanding of the structure and function of cadherin molecules is not extended significantly.

Specific Points
1) Cadherin deletion mutants were expressed in S2 cells and secreted into the culture media and analyzed by Western blotting (Fig.1). Estimated proteins and recovered proteins were different due to cleavage. In Fig.1C all DE deletion mutant molecules appear to be cleaved at NC. Then, anti-His tag antibody-conjugated beads should bind to cadherin molecules without any EC domains although some beads show Ca2+-dependent adhesion.
Based on the results, the interpretation of these experiments is difficult. It would be useful to analyze protein eluted from the beads. Also, it is possible that expression system used in this study caused cleavage of cadherins. Acquiring amino acid sequence from Drosophila embryo or adult would be direct evidence.

2) HS-AFM imaging can offer precious live information of the individual structure of molecules. In this case, glutaraldehyde-fixed molecules are imaged. Although the expressed cadherin molecules showed homophilic binding, it was not observed typically by HS-AFM. Denaturation by glutaraldehyde might cause disruption of structure for adhesion.
Also, it impels us to think that several structures shown in this manuscript may be artefacts by glutaraldehyde. Experiments elucidating structure-function relationship would resolve this problem.

3) In Fig.2, lane 17 construct should be included in E for readers to understand the experiment easily.

Reviewer 2

Advance summary and potential significance to field

Nishiguchi and Oda investigate the structural properties of the extracellular domain of Drosophila DN-cadherin in comparison to DE-cadherin, two core adherens junction proteins of the classical cadherins family.
DN-cadherin is thought to have a structure closely related to the ancestral bilaterian classical cadherin whereas DE-cadherin is a more derived form found in insects. DN-cadherin has 15 cadherin

© 2021. Published by The Company of Biologists under the terms of the Creative Commons Attribution License (https://creativecommons.org/licenses/by/4.0/).

repeats (EC) followed by membrane proximal NC, CE and LG domains. DE-cadherin has 7 EC repeats followed by NC, CE and LG domains. The 7 EC repeats in DEcad are homologous and co-linear to EC6-11 and 15 of DNcad.
Extensive structure-function data demonstrate the EC 1-11 of DNcad and EC 1-6 of DE-cad are essential for adhesion. Two different adhesion assays are used here, a bead aggregation assay with purified protein, and a cell-based adhesion assay, which both lead to similar conclusions.

The authors continue to examine the architecture of the DNcad and DEcad extracellular regions with atomic force microscopy (AFM). The evidence they collect suggest that the NC-CE-LG regions form a globular structure whereas the EC repeats are arranged in a linear fashion. EC repeats associated with Ca++ ions (hence ‘cadherins’). Ca++ binding straitens the molecule. Some EC domains lack the amino acids required for Ca++ binding suggesting that in the presence of Ca++ these positions remain flexible and can form kinks.

AFM detects one kink in DEcad that could correspond to the interface of EC2 and 3, which lacks the aa required for Ca++ binding. A conserved kink is seen in DNcad plus two additional kinks, one upstream and one downstream within the linear EC region. The bending of the DNcad and, to a lesser extent, the shorter DEcad extracellular regions could explain how these cell adhesion molecular fit into the ~20 nm intercellular space at adheres junctions.

This paper adds interesting information to our understanding of the structural parameters of cadherin adhesion molecules. The data look very clean and compelling. I think this paper would make a good addition to JCS.

Comments for the author

I have a few minor points:
1) Line 87: avoid ‘neural’ and ‘epithelial’ here. DNcad is expressed in some epithelia and DEcad is expressed also in the nervous systems including in neurons. So these terms do not make any sense here.
2) Try to reduce the number of abbreviations used to enhance the readability of the text. The names of the numerous constructs and the abbreviations of the cadherin domains are enough to keep track of. Other abbreviations are unnecessary.
3) On page 13: avoid statements “for the first time”…’provide the first…”
4) Line 527: Martin et al., is 2009 not 2008.

<u>Reviewer 3</u>

Advance summary and potential significance to field

This manuscript aims to provide a foundation for comparative structure-function studies of DN- and DE-cadherin ectodomains in cell-free systems. Using bead aggregation assays, the authors show that DN-cadherin EC1–11 and DE-cadherin EC1–6 exhibit Ca2+-dependent adhesion. Using high-speed atomic force microscopy (HS-AFM) imaging, they characterize the structures of the DN- and DE-cadherin. The scope of the manuscript is extremely ambitious and consequently the message is a little muddled. The manuscript is potentially very interesting and important to the cell-cell adhesion community. However, I have several concerns that would need to be addressed prior to publication

Comments for the author

1. Bead aggregation assays demonstrate that while DN EC1-10 and DN EC1-11 exhibit substantial levels of Ca2+-dependent aggregation, the longer DN cadherin constructs that contain EC1-11 domains do not aggregate beads. Similarly, while DE EC1-6 and DE EC1-5 exhibit s Ca2+-dependent bead-aggregation capabilities, longer DE cadherin constructs that include EC1-6 domains do not aggregate beads. This is a very confusing result and the authors make no attempt to address this in the manuscript other than offering a speculative suggestion that this may arise “due to improper orientation in representing the adhesive units on the surface of the bead or cell”. Since the bead aggregation assays are key to the messaging of the paper, addressing why additional EC domains

© 2021. Published by The Company of Biologists under the terms of the Creative Commons Attribution License (https://creativecommons.org/licenses/by/4.0/).

abrogate adhesion is critical. An immediate concern that arises is that perhaps the longer ectodomain constructs do not fold correctly into their native conformations. Have the authors tested for proper protein folding?

2. Along the same lines as the point above, from panels D and E in Figure 1, it appears that DNEXf-G has smaller sized aggregates compared to DNEXf. This suggests that adding a GFP tag interferes with adhesion in DE-cadherin. What controls do the authors have to show that appending a GFP tag does not interfere with ectodomain adhesion?

3. In the Western Blots shown in Figure 1C, why do DEEXf and DEEXf-G have the same molecular weight (when stained with DCAD2 antibody)? Shouldn't the addition of a GFP cause DEEXf-G to have a higher molecular weight? Furthermore, why are there 2 bonds in DEXf western blot that was stained with DN-Ex#8 Ab?

4. Another major issue I have is with the interpretation of the HS-AFM data. As the authors are aware, a key advantage of HS-AFM is the ability to image dynamics of molecules, often at video rates. That advantage is lost when the molecules are crosslinked to the substrate using Glutaraldehyde. This then raises the question on why use HS-AFM if dynamics are not to be measured? In Figure 5 (panels F and H), the authors apparently do image dynamic conformational changes in DN cadherin. But this then raises the question on how dynamics was imaged if the proteins were crosslinked to the substrate using glutraldehyde?

5. The analysis performed to quantify the HS-AFM images is similarly confusing. The advantage of doing an ellipse fitting is unclear since I do not see the point of calculating the long and short axis. Why not just calculate the contour length of the protein from the images since it would be much more informative? From the ellipse fitting, the authors make a big deal about quantifying variations in the long and short axis lengths and concluding that this variation is greater in DN cadherin compared to DE cadherin. I am unclear why this information is useful/surprising. Since DN cadherin is longer than DE cadherin, it follows that protein flexibility will result in a greater variation between the long and short axis for DN cadherin compared to DE cadherin.

6. Similarly, the definition of the 'SL' and 'GL' portion of the molecule in the HS-AFM image seems rather arbitrary. Since the molecules are fixed using glutaraldehyde, are the dynamic fluctuations of the arms meaningful?

7. From the height mapping data, the authors conclude that the maximum height in the GL parts was more than ~5 nm, whereas the SL parts was up to ~3 nm. What are the errors and what is the resolution of the height measurement?

8. To identify "bending sites" in the ectodomain, the authors classify the morphology of a limited number of DN cadherin ectodomains into three classes. I have serious reservations about this classification since it seems arbitrary. For instance, is a class 2 morphology really necessary? Since only 6% of DNEC14 objects exhibit class 2 morphology (and they are the only class 2 object), it seems like class 2 is potentially artifactual since it has such a limited data set (there were 149 DNEC14 objects in total which means you have just 9 objects in class 2).

9. Finally, a key ectodomain construct is missing from the classification: full length DN cadherin construct. At the very least, the authors should present data with the full length DN cadherin ectodomains (DNEXf) since they have purified this protein.

First revision

Author response to reviewers' comments

Our responses to reviewers' comments
We would like to thank all the reviewers for their constructive comments on our manuscript.

© 2021. Published by The Company of Biologists under the terms of the Creative Commons Attribution License (https://creativecommons.org/licenses/by/4.0/).

We have addressed these comments one by one as follows. We have incorporated their comments as many as possible into the new version. The changes applied to the manuscript are highlighted in blue.

Reviewer 1 Advance Summary and Potential Significance to Field:
This manuscript analyzed drosophila type III and typeIVa caderins, DN- and DE-cadherins, respectively. A number of deletion mutants were made, giving information of precursor processing of cadherins. The mutants were further used for bead aggregation assays and HS-AFM imaging. Extracellular cadherin domain repeats were highly variable, flexible in morphology with several bendable sites. These are basic features of DN- and DE-cadherins.

Reviewer 1 Comments for the Author:
This paper has shown several new findings on the Drosophila cadherins. The authors, however, did not address the mechanism of structure-function relationship of cadherin extracellular regions. Most of the data are expected or too descriptive. Our understanding of the structure and function of cadherin molecules is not extended significantly.

Response > The structural mechanisms of classical cadherin-mediated adhesion have long been explored in vertebrate systems. However, since the ectodomains of classical cadherins from nonchordate animals are quite different from those of vertebrate in terms of length and domain organization, there is little information about the structure-function relationship of such nonchordate classical cadherins. Data from nonchordate classical cadherins are essential for understanding the ancient mechanisms and origins of classical cadherin-mediated adhesion. However, there have been apparent technical difficulties in analyzing the structural and dynamic aspects of large cadherins in solution. We believe this work has made significant progress in overcoming some of these difficulties by using a combination of high-speed scan atomic force microscopy, *Drosophila* cell culture expression and purification systems, and bead-based aggregation assays. This progress has enabled us to identify the key regions of given cadherins responsible for mediating homophilic adhesion and their structural and dynamic features in solution. One of the most important findings in this work concerns the flexible hinge-like bending of the DN-cadherin ectodomain that occurs within EC6-11, the region essential for adhesion. This finding was unexpected, because bent or kinked conformations of EC repeats have been described in some non-classical cadherins but none of them have been directly visualized to be flexible or have been related to adhesion mechanisms. We believe that these findings are important in understanding the structural basis of classical cadherin-mediated adhesion in nonchordates. The data presented may help to generate hypotheses related to dynamic adhesion interfaces, responses to mechanical force, and determinants of adhesion specificities among other topics. Furthermore, comparative presentation of the datasets from two classical cadherins with distinct domain organizations in an invertebrate facilitates a better understanding of the structure-function relationship of the cadherins that accounts for evolutionary changes and diversification.

Specific Points
1) Cadherin deletion mutants were expressed in S2 cells and secreted into the culture media and analyzed by Western blotting (Fig.1). Estimated proteins and recovered proteins were different due to cleavage. In Fig.1C all DE deletion mutant molecules appear to be cleaved at NC. Then, anti-His tag antibody-conjugated beads should bind to cadherin molecules without any EC domains although some beads show Ca2+-dependent adhesion. Based on the results, the interpretation of these experiments is difficult. It would be useful to analyze protein eluted from the beads. Also, it is possible that expression system used in this study caused cleavage of cadherins. Acquiring amino acid sequence from Drosophila embryo or adult would be direct evidence.

Response > Please note that DE- and DN-cadherins are proteolytically cleaved at the NC in the extracellular region but the cleaved fragments are not physically separated from each other. Evidence from immunoprecipitation experiments in previous studies (Oda and Tsukita, 1999; Iwai et al., 1997) have demonstrated that the membrane-distal and membrane-proximal fragments are non-covalently bound to each other even after cleavage occurs in embryos and when expressed in S2 cells. In the present work, as shown in Fig. 4B (lanes 1 and 2) and Fig. S1B, S1C (lane 1), the EC-containing membrane-distal and V5-tag-containing membrane-

© 2021. Published by The Company of Biologists under the terms of the Creative Commons Attribution License (https://creativecommons.org/licenses/by/4.0/).

proximal fragments were co-purified using the V5 affinity tag. Therefore, it is most likely that the Ca2+-dependent formation of bead aggregates, shown in Fig. 1D, is mediated by EC-containing cadherin molecules.

2) HS-AFM imaging can offer precious live information of the individual structure of molecules. In this case, glutaraldehyde-fixed molecules are imaged. Although the expressed cadherin molecules showed homophilic binding, it was not observed typically by HS-AFM. Denaturation by glutaraldehyde might cause disruption of structure for adhesion. Also, it impels us to think that several structures shown in this manuscript may be artefacts by glutaraldehyde. Experiments elucidating structure-function relationship would resolve this problem.

Response > As Reviewer 1 pointed out, it is possible that denaturation by glutaraldehyde might disrupt the structure of adhesion. Although this possibility cannot be excluded, it is important to note that, as described in Materials and Methods, glutaraldehyde was added after cadherin molecules were adsorbed onto the mica and just before tip scanning began. If cadherin dimers or oligomers were present in a substantial proportion of the original solution, they could have been adsorbed onto the mica and fixed as such when glutaraldehyde was added. However, this is not the case. More importantly, we were able to observe cadherin morphologies without glutaraldehyde, which were consistently similar to (although lower in image quality than) those observed after the addition of glutaraldehyde, but there were no dimers or oligomers observed. More generally, to detect protein-protein interactions in solution using AFM imaging, the proteins must have sufficiently low dissociation constants. In response to this reviewer's comment, the manuscript has been revised to present more image data that were acquired by tip scanning without glutaraldehyde to show the consistency between cadherin morphologies obtained with and without glutaraldehyde.

3) In Fig.2, lane 17 construct should be included in E for readers to understand the experiment easily.

Response > Changed as suggested.

Reviewer 2 Advance Summary and Potential Significance to Field:
Nishiguchi and Oda investigate the structural properties of the extracellular domain of Drosophila DN-cadherin in comparison to DE-cadherin, two core adherens junction proteins of the classical cadherins family. DN-cadherin is thought to have a structure closely related to the ancestral bilaterian classical cadherin whereas DE-cadherin is a more derived form found in insects. DN-cadherin has 15 cadherin repeats (EC) followed by membrane proximal NC, CE and LG domains. DE-cadherin has 7 EC repeats followed by NC, CE and LG domains. The 7 EC repeats in DEcad are homologous and co-linear to EC6-11 and 15 of DNcad. Extensive structure-function data demonstrate the EC 1-11 of DNcad and EC 1-6 of DE-cad are essential for adhesion. Two different adhesion assays are used here, a bead aggregation assay with purified protein, and a cell-based adhesion assay, which both lead to similar conclusions.

The authors continue to examine the architecture of the DNcad and DEcad extracellular regions with atomic force microscopy (AFM). The evidence they collect suggest that the NC-CE-LG regions form a globular structure whereas the EC repeats are arranged in a linear fashion. EC repeats associated with Ca++ ions (hence ‘cadherins’). Ca++ binding straitens the molecule. Some EC domains lack the amino acids required for Ca++ binding suggesting that in the presence of Ca++ these positions remain flexible and can form kinks. AFM detects one kink in DEcad that could correspond to the interface of EC2 and 3, which lacks the aa required for Ca++ binding. A conserved kink is seen in DNcad plus two additional kinks, one upstream and one downstream within the linear EC region. The bending of the DNcad and, to a lesser extent, the shorter DEcad extracellular regions could explain how these cell adhesion molecular fit into the ~20 nm intercellular space at adheres junctions.

This paper adds interesting information to our understanding of the structural parameters of cadherin adhesion molecules. The data look very clean and compelling. I think this paper would make a good addition to JCS.

© 2021. Published by The Company of Biologists under the terms of the Creative Commons Attribution License (https://creativecommons.org/licenses/by/4.0/).

Reviewer 2 Comments for the Author:
I have a few minor points:

1) Line 87: avoid 'neural' and 'epithelial' here. DNcad is expressed in some epithelia and DEcad is expressed also in the nervous systems including in neurons. So these terms do not make any sense here.

Response > Changed as suggested.

2) Try to reduce the number of abbreviations used to enhance the readability of the text. The names of the numerous constructs and the abbreviations of the cadherin domains are enough to keep track of. Other abbreviations are unnecessary.

Response > The following abbreviations were spell out in all cases: MD (membrane-distal), MP (membrane-proximal), SL (strand-like), GL (globule-like), and PVDF (polyvinylidene fluoride).

3) On page 13: avoid statements "for the first time"…'provide the first…"

Response > Changed as suggested.

4) Line 527: Martin et al., is 2009 not 2008.

Response > Corrected as suggested.

Reviewer 3 Advance Summary and Potential Significance to Field:
This manuscript aims to provide a foundation for comparative structure-function studies of DN- and DE-cadherin ectodomains in cell-free systems. Using bead aggregation assays, the authors show that DN-cadherin EC1–11 and DE-cadherin EC1–6 exhibit Ca2+-dependent adhesion. Using high-speed atomic force microscopy (HS-AFM) imaging, they characterize the structures of the DN- and
DE-cadherin. The scope of the manuscript is extremely ambitious and consequently the message is a little muddled. The manuscript is potentially very interesting and important to the cell-cell adhesion community. However, I have several concerns that would need to be addressed prior to publication

Reviewer 3 Comments for the Author:

1. Bead aggregation assays demonstrate that while DN EC1-10 and DN EC1-11 exhibit substantial levels of Ca2+-dependent aggregation, the longer DN cadherin constructs that contain EC1-11 domains do not aggregate beads. Similarly, while DE EC1-6 and DE EC1-5 exhibit s Ca2+-dependent bead-aggregation capabilities, longer DE cadherin constructs that include EC1-6 domains do not aggregate beads. This is a very confusing result and the authors make no attempt to address this in the manuscript other than offering a speculative suggestion that this may arise "due to improper orientation in representing the adhesive units on the surface of the bead or cell". Since the bead aggregation assays are key to the messaging of the paper, addressing why additional EC domains abrogate adhesion is critical. An immediate concern that arises is that perhaps the longer ectodomain constructs do not fold correctly into their native conformations. Have the authors tested for proper protein folding?

Response > The issue of why the longer DE- and DN-cadherin constructs do not exhibit adhesion capabilities despite containing the adhesive units were addressed to some extent in our previous and present work. In our previous work (Haruta et al., 2010), we tested the capabilities of various deletion constructs for DE-cadherin in S2 cells and embryos. We showed that a DE-cadherin deletion construct in which EC1-6 was directly connected to the TM/CP region can induce cell aggregates but a DE-cadherin construct in which EC1-7 was connected to the TM/CP cannot. Importantly, both constructs produced proteins that localized to the adherens junction in epithelia when expressed in wild-type embryos. The inability of the longer DE-cadherin construct for adhesion was unlikely to be attributed to defects in protein folding and translocation following translation. In this study, AFM was used to observe the morphology of DNEC14 molecules, which did not induce bead aggregation, to compare with

© 2021. Published by The Company of Biologists under the terms of the Creative Commons Attribution License (https://creativecommons.org/licenses/by/4.0/).

DNEC11 molecules, which induced bead aggregation. However, no specific signs for defects in protein folding were found in DNEC14 molecules compared to DNEC11 molecules. What we found in this comparison was the presence of “class 2” morphology only in DNEC14 molecules, which led us to suggest that an additional bending site exists in the differential region of the DNEC11 and DNEC14 constructs. It is possible that this bending site affects the orientation of the adhesive unit. We believe that these suggestions potentially explain the incapability of the longer cadherin constructs, providing a hypothesis to be tested in the future.

2. Along the same lines as the point above, from panels D and E in Figure 1, it appears that DNEXf-G has smaller sized aggregates compared to DNEXf. This suggests that adding a GFP tag interferes with adhesion in DE-cadherin. What controls do the authors have to show that appending a GFP tag does not interfere with ectodomain adhesion?

Response > As Reviewer 3 pointed out, it is possible that the addition of a GFP tag to the cadherin ectodomain fragment might have a negative effect on adhesion capability. However, since our western blot data (Fig. 1C) showed that the amount of DNEXf-G was less than that of DNEXf, the addition of a GFP tag may have affected the efficiency of protein synthesis or the stability of the product. Therefore, we also considered the possibility that the smaller aggregates formed by DNEXf-G may be due to a lower concentration of cadherin molecules. In the bead aggregation assays, we checked and presented the amount of cadherin molecules by western blotting, in parallel. We also performed cell aggregation assays to confirm the consistency of the bead aggregation assay results using GFP-tagged constructs.
Furthermore, to minimize interference from the GFP tag, we performed an AFM-based molecular morphology analysis using GFP-free constructs.

3. In the Western Blots shown in Figure 1C, why do DEEXf and DEEXf-G have the same molecular weight (when stained with DCAD2 antibody)? Shouldn’t the addition of a GFP cause DEEXf-G to have a higher molecular weight? Furthermore, why are there 2 bonds in DEXf western blot that was stained with DN-Ex#8 Ab?

Response > Please note that DE- and DN-cadherins are proteolytically cleaved at the NC in the extracellular region (see vertical blue arrows and horizontal blue bars in Fig. 1A and B). Although the cleaved membrane-proximal fragments in DEEXf-G and DEEXf are different in size by the different tag regions, the cleaved membrane-distal fragments, which contain the epitope of DCAD2, are identical. It must also be recognized that the cleaved membrane-distal and membrane-proximal fragments are non-covalently bound to each other even after the cleavage occurs. These facts have been described in previous studies (Oda and Tsukita, 1999; Iwai et al., 1997), while the present work has added new data on the NC cleavage and another proteolytic event of DN-cadherin (Fig. S1). The two bands stained with DN-EX#8 in the DNEXf lane were cleaved membrane-distal fragments and uncleaved precursors. The corresponding upper band was very faintly detected in the DNEXf-G lane. FigS1A and S1B may help understand the cleaved and uncleaved products of the DN-cadherin ectodomain. The legend of Fig. 1 in the revised manuscript mentions the visible band corresponding to the uncleaved precursor product of DNEXf.

4. Another major issue I have is with the interpretation of the HS-AFM data. As the authors are aware, a key advantage of HS-AFM is the ability to image dynamics of molecules, often at video rates. That advantage is lost when the molecules are crosslinked to the substrate using Glutaraldehyde. This then raises the question on why use HS-AFM if dynamics are not to be measured? In Figure 5 (panels F and H), the authors apparently do image dynamic conformational changes in DN cadherin. But this then raises the question on how dynamics was imaged if the proteins were crosslinked to the substrate using glutraldehyde?

Response > It is important to note that, as described in Materials and Methods, glutaraldehyde was added after cadherin molecules were adsorbed onto the mica, just before tip scanning was started for observation. When glutaraldehyde is added to a solution containing cadherin fragments, they may form artifactual aggregates. In our experiments, however, only a small proportion of molecules in the solution were electrically bound to the mica, many others not bound to the mica are washed away, and then glutaraldehyde was added, followed by AFM

© 2021. Published by The Company of Biologists under the terms of the Creative Commons Attribution License (https://creativecommons.org/licenses/by/4.0/).

imaging. Considering that glutaraldehyde is a small molecule, crosslinking may occur between spatially close amino acid residues in a protein, causing some degree of rigidification and reducing fluctuations with minimized structural alterations. The AFM used in this study can scan images at high rates but not at video rates, which causes difficulties in imaging such dynamic molecules at sufficiently high spatial resolutions without glutaraldehyde addition. Irrespective of the glutaraldehyde addition, some surface sites of each molecule are electrically bound to the mica. If a molecule binds to mica over a wide area, it may exhibit little dynamics. Conversely, if only a part of a molecule is bound to mica, it may behave more freely and dynamically. To show, we selected molecules that happened to be in the latter situation among many molecules recorded. Importantly, the conformational variations revealed by tracking of selected single molecules were consistent with those revealed by imaging of many other molecules as snapshots, suggesting that dynamic conformational changes may account for the structural variability.

5. The analysis performed to quantify the HS-AFM images is similarly confusing. The advantage of doing an ellipse fitting is unclear since I do not see the point of calculating the long and short axis. Why not just calculate the contour length of the protein from the images since it would be much more informative? From the ellipse fitting, the authors make a big deal about quantifying variations in the long and short axis lengths and concluding that this variation is greater in DN cadherin compared to DE cadherin. I am unclear why this information is useful/surprising. Since DN cadherin is longer than DE cadherin, it follows that protein flexibility will result in a greater variation between the long and short axis for DN cadherin compared to DE cadherin.

Response > The purpose of using ellipse fitting was to investigate, in an objective way, the dimensional variations among molecules observed, which may be associated with fluctuations between bent and extended states of EC strands. However, because of the presence of the membrane-proximal globule-like portion in DNEXf and DEEXf and the difference in the polypeptide length, ellipse fitting here may be meaningless, as Reviewer 3 commented. In response to Reviewer 3's comments, we decided to restrict the use of ellipse fitting, which was only applied to DNEC14, consisting of ECs, and the shorter DN deletion constructs to assess the shape variations. In addition to the restricted use of ellipse fitting, we manually measured the lengths of individual objects for DNEXf, DEEXf, and DNEC14. These measurements were interrelated with the molecular morphologies observed by AFM (see Fig. 5 in the revised manuscript), which simultaneously responds to Reviewer 3's comment 9.

6. Similarly, the definition of the 'SL' and 'GL' portion of the molecule in the HS-AFM image seems rather arbitrary. Since the molecules are fixed using glutaraldehyde, are the dynamic fluctuations of the arms meaningful?

Response > Strand-like and globule-like morphological features of the DN- and DE-cadherin ectodomains as well as the dynamics of the DN-cadherin strand-like portion were reproducibly observed irrespective of the addition of glutaraldehyde. As shown in Fig. 6, the height profile allows us to unambiguously define the globule-like portion in most cases, although the boundary of the two portions was unavoidably ambiguous in some cases. The fluctuating behavior of DNEC14 molecules acquired in the presence of glutaraldehyde (Fig. 5G, I; Fig. S3C, D) were consistent with the dimensional variations measured from many other DNEC14 molecules at certain time points (Fig. 5E). Considering that the majority of molecules adsorbed onto the mica were less flexible in shape due to tighter binding to the mica, this consistency indicates that the shape variations are not artifacts caused by AFM imaging but likely to have existed before the binding to the mica.
In response to Reviewer 3's comments, image data showing the structural variability of the DN-cadherin ectodomain acquired without glutaraldehyde were shown in Fig. 5 in the revised manuscript.

7. From the height mapping data, the authors conclude that the maximum height in the GL parts was more than ~5 nm, whereas the SL parts was up to ~3 nm. What are the errors and what is the resolution of the height measurement?

Response > We report the height measurements in the revised manuscript. The height

© 2021. Published by The Company of Biologists under the terms of the Creative Commons Attribution License (https://creativecommons.org/licenses/by/4.0/).

resolution of HS-AFM, in general, is ~0.1-0.3 nm (Ando et al., 2008, Pflugers Arch - Eur. J. Physiol. 456: 211-225; Heath and Scheuring, 2018, Nature Comms 9: 4983).

8. To identify “bending sites” in the ectodomain, the authors classify the morphology of a limited number of DN cadherin ectodomains into three classes. I have serious reservations about this classification since it seems arbitrary. For instance, is a class 2 morphology really necessary? Since only 6% of DNEC14 objects exhibit class 2 morphology (and they are the only class 2 object), it seems like class 2 is potentially artifactual since it has such a limited data set (there were 149 DNEC14 objects in total which means you have just 9 objects in class 2).

Response > Candidly, we had difficulties in classifying the morphology of DN-cadherin ectodomain fragments. This was at least in part because there were many DNEC14 molecules showing closely folded configurations, which potentially resembled the class 2 morphology but could not be defined on objective criteria. It is possible that the class 2 morphology is not in a stable structural state, as suggested by the time sequence images shown in Fig. 5 and S3. Considering the dynamic shifts between multiple structural states and unbiased sampling, we believe that "6%" is not a small number.

9. Finally, a key ectodomain construct is missing from the classification: full length DN cadherin construct. At the very least, the authors should present data with the full length DN cadherin ectodomains (DNEXf) since they have purified this protein.

Response > In response to this comment, we manually measured the lengths of individual objects for DNEXf, DEEXf and DNEC14. These measurements were interrelated with the molecular morphologies observed by AFM, as shown in Fig. 5 in the revised manuscript.

Second decision letter

MS ID#: JOCES/2021/258388

MS TITLE: Structural variability and dynamics in the ectodomain of an ancestral-type classical cadherin revealed by AFM imaging

AUTHORS: Shigetaka Nishiguchi and Hiroki Oda
ARTICLE TYPE: Research Article

We have now reached a decision on the above manuscript.

To see the reviewers' reports and a copy of this decision letter, please go to: https://submit-jcs.biologists.org and click on the 'Manuscripts with Decisions' queue in the Author Area. (Corresponding author only has access to reviews.)

As you will see, the reviewers gave favourable reports but raised some critical points that will require amendments to your manuscript. I hope that you will be able to carry these out because I would like to be able to accept your paper, depending on further comments from reviewers.

In particular, the second reviewer has the concern about your suggestion that unlike DNEC11, the DNEC14 construct (which contains EC1-11 domains) does not aggregate beads because of an additional 'bending site' (class 2 morphology in their AFM images) which alters protein orientation. In their opinion, this suggestion is confounded by the fact that the full length ectodomain construct (DNEXf) readily aggregates beads. They feel that you need to address this apparent disparity.In addition, they recommend some additional text to be added to the discussion section.

We are aware that you may be experiencing disruption to the normal running of your lab that makes experimental revisions challenging. If it would be helpful, we encourage you to contact us to discuss your revision in greater detail. Please send us a point-by-point response indicating

© 2021. Published by The Company of Biologists under the terms of the Creative Commons Attribution License (https://creativecommons.org/licenses/by/4.0/).

where you are able to address concerns raised (either experimentally or by changes to the text) and where you will not be able to do so within the normal timeframe of a revision. We will then provide further guidance. Please also note that we are happy to extend revision timeframes as necessary.

Please ensure that you clearly highlight all changes made in the revised manuscript. Please avoid using 'Tracked changes' in Word files as these are lost in PDF conversion.

I should be grateful if you would also provide a point-by-point response detailing how you have dealt with the points raised by the reviewers in the 'Response to Reviewers' box. Please attend to all of the reviewers' comments. If you do not agree with any of their criticisms or suggestions please explain clearly why this is so.

Reviewer 1

Advance summary and potential significance to field

This manuscript shows important structural information of ectodomain of Drosophila cadherins mainly using AFM.

Comments for the author

After revision, all the points raised by this reviewer became clearer. This is a significant contribution to this research field.

Reviewer 3

Advance summary and potential significance to field

The authors have done an admirable job with their revisions and have satisfied most of my concerns. However, I continue to have one serious concern that the authors still need to address. I also have a suggestion that will hopefully help them enhance the reach of this paper.

Comments for the author

Concern: The author's suggest that unlike DNEC11, the DNEC14 construct (which contains EC1-11 domains) does not aggregate beads because of an additional 'bending site' (‘class 2 morphology’ in their AFM images) which alters protein orientation. However their explanation is confounded by the fact that the full length ectodomain construct (DNEXf) readily aggregates beads. What happened to the kink region in the full length construct? Did they observe any class 2 morphology with the DNEXf constructs? If not, why not? The authors need to address this apparent disparity.

Suggestion: The author's explanation for the smaller DNEXf-G bead aggregates compared to DNEXf bead aggregates seems reasonable. However, I would recommend that they add text to the discussion section clearly articulating this (like they did in the response letter). Readers are undoubtedly going to be confused by this point as I was.

Minor comment: On line 346 of the manuscript, replace ‘DEEC14’ with ‘DNEC14’

Second revision

Author response to reviewers' comments

Our responses to reviewers' comments
We would like to thank all the reviewers for their constructive comments on our manuscript. We have addressed these comments one by one as follows. We have incorporated their comments as

© 2021. Published by The Company of Biologists under the terms of the Creative Commons Attribution License (https://creativecommons.org/licenses/by/4.0/).

many as possible into the new version. The changes applied to the manuscript in this second revision are highlighted in red.

Reviewer 1 Advance summary and potential significance to field
This manuscript shows important structural information of ectodomain of Drosophila cadherins mainly using AFM.

Reviewer 1 Comments for the author
After revision, all the points raised by this reviewer became clearer. This is a significant contribution to this research field.

Response> Thank you for understanding the significance of our work.

Reviewer 3 Advance summary and potential significance to field
The authors have done an admirable job with their revisions and have satisfied most of my concerns. However, I continue to have one serious concern that the authors still need to address. I also have a suggestion that will hopefully help them enhance the reach of this paper.

Reviewer 3 Comments for the author
Concern: The author's suggest that unlike DNEC11, the DNEC14 construct (which contains EC1-11 domains) does not aggregate beads because of an additional 'bending site' (‘class 2 morphology’ in their AFM images) which alters protein orientation. However their explanation is confounded by the fact that the full length ectodomain construct (DNEXf) readily aggregates beads. What happened to the kink region in the full length construct? Did they observe any class 2 morphology with the DNEXf constructs? If not, why not? The authors need to address this apparent disparity.

Response> Thank you for your comments on this important matter.
To explain why the DNEC14 construct and some longer ones do not aggregate beads despite having the EC1-11 region, we suggest possible negative effects of a bending site localized in the differential region of the DNEC11 and DNEC14 constructs on binding ability. In this suggestion, we assume that the negative effects may be due to improper orientation in representing the adhesive unit on the surface of the bead or cell. After this reviewer's comment, however, we realized the need to explain about the consistency with the normal situation. The negative effects caused by the addition of ECs (eg., EC12-EC14) were neutralized by further addition of the membrane-proximal domains. Therefore, in addition to the suggestion of improper orientation in representing the adhesive unit, we consider that the ECs that are more membrane-proximal than the adhesive unit might have interactions with the following non-EC domains to constitute another functional and/or structural unit. The lack of a part, not the whole, of this unit might produce a negative effect on the performance of the adhesive unit. These possibilities could not be investigated due to technical limitations in the present work but should be addressed in future studies.

Since our AFM-based analyses of DNEXf molecules, due to technical limitations, have not allowed us to determine the relationship between the domain positions and imaged morphologies, we cannot answer the questions.

To explain the possibilities we consider, we rewrote the related part of the discussion (p.16).

Suggestion: The author's explanation for the smaller DNEXf-G bead aggregates compared to DNEXf bead aggregates seems reasonable. However, I would recommend that they add text to the discussion section clearly articulating this (like they did in the response letter). Readers are undoubtedly going to be confused by this point as I was.

Response> Thank you for your suggestion. To explain this, we added a new paragraph in the discussion (p. 15).

Minor comment: On line 346 of the manuscript, replace ‘DEEC14’ with ‘DNEC14’.

Response> Corrected.

© 2021. Published by The Company of Biologists under the terms of the Creative Commons Attribution License (https://creativecommons.org/licenses/by/4.0/).

Third decision letter

MS ID#: JOCES/2021/258388

MS TITLE: Structural variability and dynamics in the ectodomain of an ancestral-type classical cadherin revealed by AFM imaging

AUTHORS: Shigetaka Nishiguchi and Hiroki Oda
ARTICLE TYPE: Research Article

I am happy to tell you that your manuscript has been accepted for publication in Journal of Cell Science, pending standard ethics checks.

© 2021. Published by The Company of Biologists under the terms of the Creative Commons Attribution License (https://creativecommons.org/licenses/by/4.0/).
